# Advanced nursing practice for caring for mothers of children with autism spectrum disorder in the CACTO program^*^

**DOI:** 10.1590/1518-8345.8152.4882

**Published:** 2026-07-24

**Authors:** Paulo Roberto Lima Falcão do Vale, Evany Caroline de Souza Cerqueira, Deisy Vital de Melo, Ana Paula Dezoti, Verônica de Azevedo Mazza, Rosely Cabral de Carvalho, Evanilda Souza de Santana Carvalho

**Affiliations:** 1 Universidade Federal do Recôncavo da Bahia, Centro de Ciências da Saúde, Santo Antônio de Jesus, BA, Brazil.; 2 Universidade Estadual de Feira de Santana, Departamento de Saúde, Feira de Santana, BA, Brazil.; 3 Centro Universitário Autônomo do Brasil, Departamento de Enfermagem, Curitiba, PR, Brazil.; 4 Universidade Federal do Paraná, Curitiba, PR, Brazil.; 5 Scholarship holder at the Conselho Nacional de Desenvolvimento Científico e Tecnológico (CNPq), Brazil.

**Keywords:** Advanced Practice Nursing, Autism Spectrum Disorder, Caregivers, Primary Care Nursing, Nursing Theory, Evidence-Based Nursing

## Abstract

**Objective:**

to describe the competencies of advanced nursing practice for the care of mothers participating in CACTO: a unitary care program for mothers of children with autism spectrum disorder and/or disability.

**Methods:**

qualitative study based on the Unitary Caring Science, conducted with 39 mothers of children with autism spectrum disorder. Data were collected from medical records, field diaries, meeting minutes, and CACTO’s social media profile. Deductive, reflective thematic analysis was used for data analysis.

**Results:**

the results revealed a broadening of the scope of competencies that can be performed by nurses in primary health care, for example: understanding health needs, creating new ways of caring, recognizing ethical dilemmas, establishing collaborative relationships, valuing the social role of mothers, suggesting care with recommended scientific evidence, conducting scientific research, and acting autonomously.

**Conclusion:**

the competencies of nurses working at CACTO are distributed across seven dimensions, namely: care management, ethics, interprofessional collaboration, health promotion and risk prevention, evidence-based nursing, research, and leadership.

## Highlights

**(1)** The skills of nurses working in CACTO can broaden the scope of practice in PHC. **(2)** The creation and invention of new ways of caring can increase the effectiveness of PHC. **(3)** PHC nurses should seek new solutions for mothers’ health needs. **(4)** User involvement is essential for the implementation of care technologies. **(5)** PHC nurses should invest in subjectivity and abstraction skills.

## Introduction

The prevalence of Autism Spectrum Disorder (ASD) has increased significantly; it is estimated that approximately 1 in 31 children worldwide have ASD^1^. In Brazil, approximately two million people live with the disorder^2^. Mothers play a key role in recognizing their children’s disabilities, managing their behavior, seeking appropriate therapies, and developing the therapeutic plan necessary for their children’s development^3^. In this scenario, mothers frequently access medium-tech health facilities^4^, such as Specialized Rehabilitation Centers (CER, in Portuguese), Whereas few care initiatives are directed at mothers in Primary Health Care (PHC)^3^.

Given the burden of care, high levels of anxiety and depression, poor quality of life and sleep, as well as low adherence to self-care and interruption of their life plans in pursuit of their child’s autonomy^3^, mothers live with risk factors that place them in a vulnerable situation. Given health needs and the lack of care, it is up to PHC to provide care that promotes the health and emancipation of mothers, take measures to confront hegemonic heteronormativity, and reorganize the Health Care Network (RAS) into an inclusive continuum of care for the mother-child dyad.

Anchored in the attributes of bonding, health accountability, longitudinality, and capillarity, specific care for mothers implemented in PHC is advocated. Therefore, it is essential to develop programs that meet the health needs of mothers. In this sense, CACTO [acronym in Portuguese for Care Program for Mothers of Children with Congenital Zika Syndrome^5^] was developed and subsequently applied to care for mothers of children with ASD^6^.

CACTO, based on the Unitary Caring Science (UCS) proposed by nurse Jean Watson^7^, enables innovative care with a humanitarian approach that contrasts with exclusionary and individualistic practices, thereby promoting more comprehensive, equitable, and emancipatory care^6^. It acts on the health repercussions experienced by mothers through a set of specific care measures to promote well-being, reduce vulnerabilities, enhance human existence, and restore (healing)^5^.

Gaps in knowledge and decontextualized, repetitive practices result in ineffective nursing care that is sometimes inadequate for the needs of people in vulnerable situations^8^. In this sense, Advanced Practice Nursing (APN) emerge as an important construct aimed at ensuring nurses’ practice, primarily by qualifying their clinical practice in PHC^9^.

APN refers to the work of nurses, based on specialized knowledge, complex decision-making skills, and expanded clinical competencies, which give them greater professional autonomy and enable them to address the unique cultural and regulatory aspects and specific needs of the populations they serve^10^. The Advanced Practice Nurse (APN) expands their clinical scope and assumes strategic roles in leadership, care management, education, and research, articulating these dimensions to qualify care with a focus on safety, effectiveness, innovation, and health promotion^10^.

In clinical practice, the (APN) seeks to respect the uniqueness of the individual, family, and community, acting as a transformative agent in health services. Their academic training is advanced and continuous, which supports improved clinical reasoning, critical use of the best scientific evidence, and an ethical commitment to risk prevention, interprofessional collaboration, care management, professional autonomy, leadership, and technological mastery, promoting the constant evolution of nursing^10^, skills necessary for the uniqueness and quality of care in PHC, as well as increasing resolution rates.

In caring for families of children with ASD, nurses have offered emotional support, provided guidance on practices to alleviate the burden of care, coordinated interprofessional interventions^11^, identified early signs of ASD, and coordinated the therapeutic pathway^12^. However, there is little evidence of APN for mothers of children with ASD and/or disabilities.

The process of implementing APNs should be guided by scientific evidence that supports EPA (APN) practice, such as skill sets organized into domains, namely: care management, ethics, interprofessional collaboration, promotion and prevention, evidence-based nursing, research, and leadership, directly aligned with the guidelines, supporting EPA(APN) practice^9^. This poses the challenge of training, qualifying, and developing nurses’ skills for the longitudinal care of people in vulnerable situations, as well as valuing users as protagonists of a culturally appropriate technology such as CACTO.

Given the need for innovative care models implemented by nursing in PHC, as well as the need to regulate the competencies for EPA (APN) training in Brazil, this study aims to answer the following research question: What APN competencies are applied to the care of mothers of children with autism spectrum disorder participating in the CACTO program? The objective of this study is to describe the competencies of advanced nursing practice for the care of mothers participating in the Program.

## Methods

### Type of study

Qualitative study based on the implementation o f the CACTO program grounded in the Unitary Caring Science^7^ as a theoretical framework. This study was written in accordance with the Consolidated Criteria for Reporting Qualitative Research (COREQ) guidelines.

### Study location and data collection period

This study was developed within the scope of the CACTO program, which began in October 2023 and continues to operate at the TEAbraco SAJ institute to date. Data collection for this manuscript was conducted between October 2023 and January 2025.

The institute is a non-governmental organization run by mothers of children with ASD, maintained through donations and voluntary partnerships, which offers physical therapy, psychology, speech therapy, neuropsychopedagogy, music therapy, and social assistance services exclusively for children. Access to therapeutic services is provided through direct payment by families; in specific cases, following a social analysis, some families benefit from free access to services. Approximately 260 families are associated with the institute, located in the municipality of Santo Antônio de Jesus (BA), which has a resident population of 103,055, a municipal human development index of 0.700, and a gross domestic product of R$ 23,746.79.

### Theoretical framework

In 1979, Jean Watson analyzed human care, focusing on the mode of interaction between nurses and those receiving care, which was increasingly distancing itself due to the introduction of hard technologies, placing interactional care in the background. This led to the emergence of the Theory of Human Caring and the Carative Factors as the first paradigm, emphasizing the moment of caring as an end rather than a means to achieve healing^7^.

UCS is Watson’s current theoretical construct, based on the Unitary-Transformative paradigm, which holds that all beings are interconnected by universal cosmic energy, a source of life that is love, and the starting point of the ontology of being. Interaction with each person requires the nurse to raise their consciousness, be genuinely present, be open to giving and receiving, apply moral and ethical values, and use nursing skills, attitudes, and knowledge as a science that fosters transcendence^7^.

Seeking to promote nursing literacy on UCS, Watson developed *Veritas*, represented by a vibrant word that emanates positive energy and life. *Caritas*-*Veritas* care is grounded in love as the source of life and in the evolution of consciousness and intentionality to achieve healing (*Caritas*), thereby ascribing to each *Caritas* its moral component and ethical value applied in the practice of care (praxis)^7^.

The care meetings developed in the CACTO program are based on UCS^7^, respecting the ten elements of the Clinical Process *Caritas*-*Veritas* (PCC-V), valuing the mother’s free narrative, avoiding speech restriction, and guiding the process toward self-understanding and the planning of self-care practices. The ten elements of the PCC-V are: Love-Kindness; Faith-Hope; Self-Transpersonal; Nurturing-Relating; Forgiving; Self-Creating; Learning; *Caritas* Environment; Humanity; Infinity^7^.

**Figure 1:**
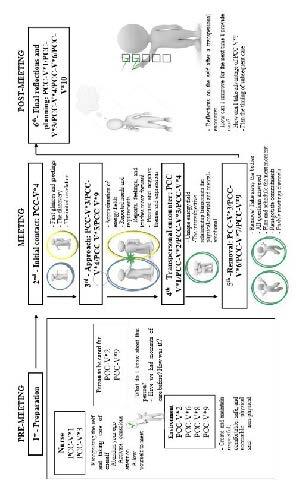
Six steps for conducting a care meeting based on the Unitary Caring Science

At CACTO, the care encounter between nurse and mother occurs in six steps, when it is possible to apply each element of the PCC-V as shown in Figure 1:

*PCC-V = *Caritas*-*Veritas* Clinical Process

### Participants

Self-identification as the mother of a child with ASD was the only inclusion criterion for this study. As this group of women has needs that are rarely addressed by health services and given that CACTO could benefit a significant number of mothers, and that no variables would make the sample heterogeneous, no exclusion criteria were defined.

Forty-four mothers who attended the TEAbraco SAJ Institute during CACTO’s weekly operating hours were invited to participate. There were three refusals justified by lack of time, one due to chemotherapy treatment, and another due to disagreement with the spouse, resulting in a final sample of 39 participants, identified by codes such as M1, M2, M3..., M51.

### Data collection instruments

The data sources were medical records, completed after the care meeting, field diary (organized in a file with the following sections: description of the event, inference, what to do?), and meeting minutes, documents prepared by the nurse and stored on Google Drive, in addition to data extracted from CACTO’s Instagram profile (@cacto.ufrb). Care meetings were audio-recorded, transcribed, and imported into individual medical records. The medical record was prepared in accordance with the UCS, the Problem-Oriented Clinical Record model, the Subjective, Objective, Assessment, Plan method, and the International Classification of Primary Care, 2nd edition (ICPC-2). Appendices to the medical record include notes, agreements, the care plan and interventions, the referral and counter-referral form, and the longitudinal care spreadsheet.

The medical record is structured in ten sections, namely: sociodemographic and economic identification of the mother and child; who are you? current life history; personal history; family history; history of ASD in the child; social support and relationship with the child; self-care and lifestyle habits; family and social aspects (genogram and ecomap); final remarks and reflections.

### Data collection

After obtaining authorization from the institute’s management, the CACTO nurse first spoke with the mothers individually in the reception area of the institute’s headquarters, where the nurse invited them to participate in the program. The 39 mothers were invited to visit the CACTO facilities, located at the institute’s headquarters. Next, the informed consent form and the image and sound use authorization form were read and signed. The mothers were informed that the data produced from the care meetings would be used for research purposes.

The nurse working at CACTO has more than 11 years of training, including a Bachelor of Nursing degree and a specialization in Family Health, with a focus on multi-professional health residency. He is a specialist in Health Management, holds master’s and doctoral degrees in Public Health, and is trained in various integrative and complementary practices. He was a scientific initiation fellow in studies with families for three years during his undergraduate studies. Throughout his professional career, he has worked in the Family Health Strategy (FHS) and as a teacher, teaching related subjects in primary care and public health, and developed CACTO during his PhD in Public Health (2018-2022).

The role of nurses working at CACTO includes: organizing care appointments; preparing the care environment; documenting information in medical records; developing care plans; coordinating care through individualized therapeutic projects; training staff through continuing education sessions; and raising funds for the program’s operations and the care of mothers.

To ensure the reliability of data extraction, the authors met in advance to understand the domains and competencies of the EPA (APN); then, a matrix was developed with the seven domains of the EPA (APN) in rows and the four data sources of the research arranged in columns. After resolving epistemological and methodological questions, the first and second authors simultaneously filled out the matrix in Microsoft Office Word 2019 software between January and February 2025. To achieve a broad coverage of the identified competencies, all data aligned with the APN domains were included, ensuring agreement among the authors when completing the matrix.

### Data analysis

Reflective deductive thematic analysis (TA)^13^ was employed using seven EPA (APN) competency domains chosen as the lens of analysis, namely: care management, ethics, interprofessional collaboration, promotion and prevention, evidence-based nursing, research, and leadership^9^. The first stage involved deepening the theoretical body, conducted prior to data extraction, through exhaustive reading, meetings, and scientific sessions on the EPA (APN) domains^9^. This was followed by the six phases of TA: familiarization with the data; coding, when the authors searched for codes that met the competency domains; due to the large amount of data, a second round of coding was necessary, resulting in 68 codes; in the initial theme generation phase, the data extraction and analysis matrix with the 68 codes was made available on Google Drive for all authors to contribute. After seven days, the authors met to suggest revisions to the codes and to group them by similarity, context, or chronological order, resulting in 38 themes, or 38 nursing competencies. Existing conflicts were resolved by consensus among all authors. In the fourth phase, the first and second authors returned to the database to identify convergent and divergent data for consolidation of the themes. No data was added or removed in this phase. During the phase of refining, defining, and naming themes, the competencies were revised and presented at a discussion and reflection meeting, with all authors in attendance, to elicit potential inferences; and finally, the last phase consisted of the final draft.

### Ethical considerations

The study was approved by the Human Research Ethics Committee of the Federal University of Recôncavo da Bahia with Ethical Review Certificate No. 65573422.5.1001.0056, opinion number 5,866,634.

## Results

Thirty-nine mothers participated, aged between 36 and 40 (33.3%) and between 41 and 45 (20.5%), with 16 (41%) married and 19 (48.7%) identifying as black and 18 (46.1%) as brown. Twenty participants (51.3%) had completed high school, 15 (38.5%) were housewives, 16 (41%) reported having no income, and 14 (36%) had an income between R$ 600.00 and R$ 1,518.99. The age of children with ASD reported by their mothers was predominantly 4 to 5 years old, distributed among 14 children (36.9%), and 11 (28.2%) were between 6 and 12 years old.

The competencies of nurses applied to the quality of care for mothers of children with ASD at CACTO, categorized by domain, are described in Figure 2. 

**Figure 2:** APN* domains, competencies of nurses working in the CACTO program, and empirical evidence

Domains	Competencies	Empirical evidence
**Care management**	**Care approach** Understands the main health needs of mothers.	Assignment: Write a letter describing what today’s M12 would write to M12 when she discovered her pregnancy.
	Apply for UCS^†^.	
	**Assessment and diagnosis** Explores dialogue in a broad and comprehensive manner.	Medical record entry: *People say, “Being a mother is not an easy task.” I knew it wasn’t, but I didn’t know I would be alone, [...] in the desert, just me and my son, because all the doors are closed* (M11).
	Conducts investigative and in-depth anamnesis considering history, trauma, and significant past experiences.	
	Uses audio recorders to ensure accurate data capture and database construction.	
	Applies instruments to measure levels of anxiety, depression, hope, resilience, fatigue, caregiver burden, and quality of life.	
	Standardizes diagnosis based on ICPC-2^‡^, recommended by the Brazilian Ministry of Health.	Medical record entry (main problems): Z12 - relationship problems with spouse; P01 - feelings of anxiety/nervousness/tension; T07 - weight gain.
	**Care provision** Develops and reassesses PTS^§^ together with the mother.	Medical record entry: *I made the agreement. It worked. I went to my father’s house less often, went out more on my own, did things with my children. At my father’s house, I feel very overwhelmed* (M56).
	Provides emotional support, active listening, and therapeutic touch/hugs.	
	Provides guidance on physiology, expected behaviors, and interacting with children with ASD^ǁ^.	
	Guides mothers regarding RAS^¶^ flows.	
	Performs integrative and complementary practices.	Post published on CACTO’s profile. Available via link.: https://www.instagram.com/p/C6MZ1wFAEr8/?igsh=MXVheHc3eHZtc2p1Yw==
	Prescribes the use of herbs and teas, as well as recommending the continuation of commonly used style="padding:10px; border:1px solid black" allopathic medications.	Prescribed agreement for M51: Consume *mulungu* tea (3g/300ml of water). Use: four 300ml cups per day, prepared by decoction (boil with lid open).(M51).
	Implements care agreements, such as: “appreciating silence”, “valuing the presence of the self”, and “performing contemplation exercises”.	Medical record*: I made the agreement, slept better, prepared the environment, took away my cell phone, and it worked* (M01).
	Prescribes the care modalities provided for in CACTO.	Agreement prescribed for M23: Practice the primer by taking care of my mind for 21 days. Remember to access the available podcasts*.*
	Creates new ways of caring based on mothers’ needs.	Intervention sheet: “Draw something that represents the ideal woman” (Figure 3).
**Ethics**	Builds a therapeutic environment that preserves confidentiality, privacy, and comfort.	Posts published in the “*Ambiência*” highlight on CACTO’s profile. Available via link: https://www.instagram.com/s/aGlnaGxpZ2h0OjE3OTMwOTM1ODMyOTY5NzUx?story_media_id=3256359463652818529_61260366393&igsh=b2VuYXgyN3p3N2wz
	Integrates the child’s presence during care.	
	Respects the ethical principles of bioethics, human rights, and professional ethics.	Medical record: *I didn’t want to go back to the countryside, but his father wanted to, so I came. I know he cheats on me, he has other women, but I don’t want my son to grow up without a father* (M41).
	Recognizes the ethical dilemmas faced by mothers, such as separating from the father and “raising the child without a father” or enduring situations of violence and infidelity.	
**Interprofessional collaboration**	Discusses cases with professionals from the multidisciplinary team.	Meeting minutes: On the fourteenth day of November 2024, the institute’s healthcare team met to discuss the progress of the therapeutic process of (name omitted) and his mother M41. [...]
	Establishes a collaborative relationship with the institute’s management.	
	Complete a referral form to share cases with RAS^¶^ professionals.	
**Promotion and prevention**	Encourages mothers to recognize their qualities, value themselves, and prioritize their own needs.	Prescribed agreement for M44: What are my strengths and challenges?
	It values the social role played by women.	
	Encourages the adoption of healthy eating habits, reduced style="padding:10px; border:1px solid black" alcohol consumption, and the reduction of sedentary behavior.	Prescribed agreement for M10: Replace one of the snacks with fruit of your choice. Eat together with fiber: flaxseed, *chia*, oatmeal, or others.
	Develops informational materials to combat ableism and disseminates them via social media.	
**Evidence-based nursing**	Builds and uses evidence-based care modalities.	Development of the “taking care of my sleep” care modality^5^, based on the guidelines of the Brazilian Sleep Association. Prescribed agreement for M1: start the step-by-step process of taking care of my sleep.
	Proposes goals in the STP^§^ based on clinical evidence.	
	Adheres to protocols established by the Brazilian Ministry of Health.	
**Research**	Develops and guides research to assess phenomena experienced by mothers.	Posts published on CACTO’s profile. Available via link: https://www.instagram.com/p/C6KbRzVgN0F/?igsh=Znk5ejJoNXVtbTQ=
	Develops research project for the expansion of CACTO based on a call for proposals from the Brazilian Ministry of Health.	
	Conducts scientific sessions with partner research groups.	
**Leadership**	Acts independently and with authority.	Record in medical records: the frequency and scheduling of each mother’s appointments are recorded in the longitudinal spreadsheet.
	Defines the frequency of consultations based on each mother’s needs.	
	Schedule the mother’s appointment at the same time as the child’s appointment.	
	Promotes collective care strategies through the Experiences Group and the Focus Group technique.	Field Diary of the Experiences Group held on 08/14/24: Mothers share experiences on what to do when someone criticizes their child.
	It coordinates with the Family and Community Medicine Residency and professors from the bachelor’s degree program in Nursing and Medicine.	Record in medical records: Referral form completed and sent to the referring physician.

*APN = Advanced Practice Nursing; ^†^UCS = Unit Care Science; ^‡^CIAP-2 = International Classification of Primary Care; ^§^STP = Singular Therapeutic Project; ^||^ASD = Autism Spectrum Disorder; ^¶^RAS = Health Care Network

**Figure 3:**
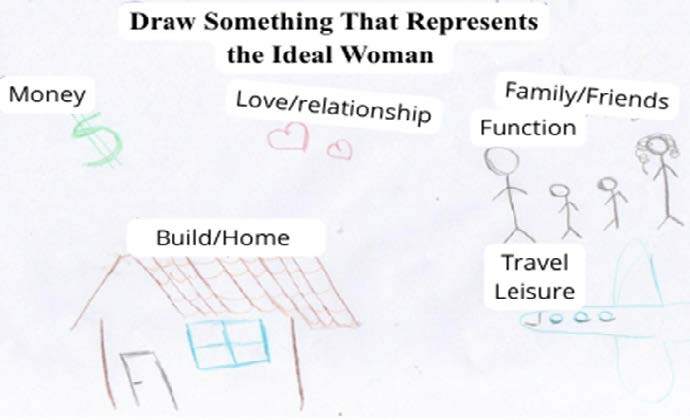
Drawing made by M45 after intervention created by the nurse and stimulated by the phrase: “Draw something that represents the ideal woman”

## Discussion

The results of this study broaden the scope of competencies that nurses can perform in PHC^14^ and provide scientific evidence supporting nursing care practices aimed at the well-being of mothers, with positive repercussions for children with ASD and other family members. In this scenario, public policy managers and implementers can choose to train PHC nurses to develop and implement care guidelines to increase the accessibility of universal health systems, improve the effectiveness of PHC, reduce inequalities, enhance quality of life, and lower costs^15^.

Many countries lack structured care programs capable of integrating early diagnosis, continuous multidisciplinary interventions, and longitudinal support for families and individuals with ASD^16^. This scenario reinforces the need for specific programs coordinated by nurses, considering that most existing models focus solely on the diagnostic stage, without ensuring adequate follow-up or support for the emotional and socio-educational demands of the family unit^17^.

At the time of writing this article, there were no programs or lines of care directed at mothers of children with ASD, nor were there any APNs based on nursing theories^18^, such as UCS. Therefore, care management at CACTO is carried out by the program’s coordinating nurse, who encourages mothers to take a leading role, considering the focus on care, assessment, diagnosis, and care provision^9^.

The first stage of CACTO development is called “understanding”^5^; it involves understanding the health needs of mothers, so it is important for nurses to access a wide range of scientific literature with high levels of evidence and recommendations, as well as gathering essential information through home visits. Therefore, before the implementation of CACTO, considering the subdomain of care focus, it was known that mothers needed access to social protection rights, goods, and services such as housing and basic sanitation, food security, work, and schooling; qualified services and empathetic multidisciplinary care; welcoming, strengthening bonds, and acceptance; parental accountability and sharing of daily responsibilities and care of the self^5^.

The focus of care at CACTO is based on the UCS, seeking the healing of the living being through the balance among the body-mind-soul dimensions that occurs during and after transpersonal care^7^. The nurse working at CACTO developed an exchange period during his doctorate, with the aim of mastering UCS, an experience that contributed to planning the applicability of PHC attributes during the implementation of CACTO, such as territorialization, bonding, social participation, health accountability, and user protagonism^18^.

Extensive and exhaustive dialogue was the technique used during the anamnesis to better understand the risks and problems that determine the process of illness in mothers. The concept of people as historical subjects, constituted by the experiences that shape their beliefs and behaviors^19^, justifies the need for investigative and in-depth anamnesis, with data collection from birth to the present, identifying the traumas, sorrows, pleasures, and joys experienced. At this point, it is appropriate to value the work process of the mother’s Community Health Agent and their contribution to the development of users’ STP^20^.

Assessment and diagnosis in APN is a continuous process that involves the collection and analysis of objective and subjective data. This allows for the review of diagnoses, problem lists, care plans, interventions, and strategies based on the patient’s role and their support network^18^. In the diagnostic process, the EPA should use clinical assessment data assigned to standardized clinical classification systems^21^ such as ICPC-2, which is used in CACTO.

The EPA interested in applying UCS^7^ must exercise unique skills such as the ability to abstract, understand the totality of the sacred human being, recognize what is said and unsaid, expressions and silences, control professional and personal impulses that hinder the thinking of those in care, creativity to create interventions (Figure 1) and agreements, as well as agility to implement them at opportune moments.

The ethical dilemmas experienced by nurses at CACTO are complex and require delicate and prudent decision-making based on the health needs and priorities of mothers. These experiences include choosing between separating from a spouse and ending situations of violence or remaining in the marital relationship due to financial dependence; or resuming professional goals and feeling guilty for outsourcing childcare or continuing to care for the child full-time and living with financial difficulties. Nurses’ ethical decisions are informed by professional legislation, institutional protocols, and the Universal Declaration on Bioethics and Human Rights, as well as by the principle of moral sensitivity, which enables them to recognize the ethical dimensions of problems and to involve the patient in the decision-making process^22^.

Interprofessional collaboration fosters a culture of mutual respect, trust, and recognition for the contributions of each team member, creating an environment conducive to innovation, creativity, and excellence in care. In this way, it enhances knowledge sharing, improves care and decision-making, increases patient safety, and optimizes resources^23^. Care for mothers requires compliance with the functions of PHC as coordinator of care and organizer of the RAS to optimize response time, shorten therapeutic itineraries for themselves and their children, and increase resolution based on the possibilities of matrix support with the multidisciplinary team in PHC (eMulti) and the therapists of the children who make up the RAS. The principle of intersectorality must also be coordinated by the PHC team to meet the health needs of mothers at the Reference Centers for Social Assistance (CRAS) and schools/daycare centers attended by their children.

The needs of mothers manifest as emotional, behavioral, psychosocial, and clinical-biological problems that can be understood as domains of the body, mind, and soul^17^. The assessment and diagnosis indicate that nurses working at CACTO should explore skills in managing the care of chronic conditions; therefore, actions to prevent risks and harm and promote health are a priority. Empirical evidence on compliance with agreements made at CACTO points to high satisfaction among mothers, and other studies included in a systematic review indicate that EPA has a positive impact on patients’ quality of life^24^.

The management of chronic conditions is guided by self-esteem, recognition of one’s potential and limitations, awareness of one’s health-illness process, alignment of expectations regarding cure, degree of self-determination, coping, and motivation^25^. The attributes and functions of the FHS, such as accessibility, bonding, welcoming, humanization, territorialization, health accountability, coordination, and longitudinality of care^26^ were elements applied by the CACTO nurse and contributed to revealing the hidden demands of mothers, increasing the rate of attendance at consultations, promoting adherence to the agreements implemented, and favoring the social appreciation of the maternal role and self-esteem.

The variety of diagnoses and the unique, personalized nature of CACTO required implementing a broad scope of care and developing new evidence-based care. The EPA must develop investigative skills to find the best scientific evidence and clarify questions that arise in the field of practice. Research skills contribute to innovation through the development of new research, the development of health promotion protocols, and the implementation of new care technologies in PHC^9^.

It is also worth noting, based on this experience, the fundamental role of users in the development of culturally appropriate technologies. The diversity and broad scope of the CACTO nurse’s skills endorse the relevance of *stricto sensu* courses for improving technical products, encouraging the development of social technologies, epistemological deepening, and applicability of nursing theories, as well as the management of care programs, nursing as a science, and the implementation of APN in PHC.

Nurses working in PHC need to demonstrate characteristics such as commitment to the profession, critical and reflective analysis, capacity for abstraction, and educational and clinical skills consolidated by qualified training in teaching and research. These aspects enable them to practice clinically across a variety of diagnoses, provide diverse care, coordinate care plans, promote longitudinal care, and express autonomy and authority, qualities expected of a Nurse Practitioner^14^ *.*

The results indicate the strengthening of the professional identity of CACTO nurses, by enhancing their clinical reasoning and leadership role in the interprofessional team^27^ and providing solutions to critical issues that remain unanswered from the implementation of programs, policies, or practices aimed at producing impacts on access and inequalities for people in vulnerable situations. The evidence from this study highlights the quality of nursing education, underscores the relevance of PHC, and the need for additional training in APN at the postgraduate level, with reservations regarding the regional specificities of each country.

Findings from scope review studies have revealed that the performance of the EPA in PHC is associated with fewer patient visits to emergency rooms, lower incidence of hospital admissions or readmissions, fewer consultations in PHC, greater adherence to care plans, and lower health-related costs^28^. The training of EPA through doctoral courses in nursing practice with an emphasis on PHC is an initiative that is expanding around the world^29^.

Ethnography conducted with nurses in Switzerland identified the following functions of the EPA in PHC: defining the functions and operation of the health team, resolving interprofessional conflicts, person-centered care, collaborative leadership, and interprofessional communication^30^. In the international context, EPA has contributed to expanding access to PHC, offering care conducive to active aging, and managing care for people with chronic diseases^31^.

In the United Kingdom, EPAs and family nurses working in the community develop similar skills, with EPAs playing a greater role in research, while family nurses often work in the areas of education and leadership^32^. However, several studies agree that the legal particularities and territorial needs of each country should define the scope of EPA activities^28^ ^-^ ^30^.

This study contributes to the advancement of scientific knowledge by presenting innovative initiatives for caring for a rapidly growing population with health needs that are largely unexplored in health services: mothers of children with ASD. It is understood that the identified skills can support nurses in providing care aligned with mothers’ needs. The results enable discussion of the competencies performed by CACTO nurses and those applied by PHC nurses worldwide; therefore, future studies may examine and evaluate the quality of care for mothers of children with ASD and the PHC nurse training process, with consideration of mothers’ health needs.

The limitations of the study include the specific characteristics of the TEAbraco SAJ institute, grounded in the principles of association, voluntarism, and solidarity, which may have influenced motivational factors, decision-making processes, and intersubjective relationships between nurses and mothers. Accordingly, the evidence obtained should be applied with caution in other RAS care settings.

## Conclusion

The competencies of nurses working at CACTO are organized into seven dimensions, namely: care management, ethics, interprofessional collaboration, promotion and prevention, evidence-based nursing, research, and leadership. Among these competencies, the following stand out: the creation and invention of new ways of caring, autonomy in caring for mothers, prudence in ethical dilemmas, investigative and research skills, as well as leadership and protagonism for coordinating care and organizing the RAS, to expand the scope of practice in PHC and increase its effectiveness.

The nurse working at CACTO performed tasks that can be applied by nurses in primary health care based on strategies to expand the scope of nursing practices, such as promoting self-care among mothers, contributing to the psychosocial and cognitive development of children, empowering parents, and acting politically in favor of the social inclusion of families with children with ASD.

## Data Availability

All data generated or analysed during this study are included in this published article.
