# Supplementary material for: Advanced nursing practice for caring for mothers of children with autism spectrum disorder in the CACTO program
Source: Rev Lat Am Enfermagem. 2026 Jul 24;34:e4882. doi: 10.1590/1518-8345.8152.4882 (PMC13405589; doi:10.1590/1518-8345.8152.4882)
Supplement: Translation

# Translation: Prática avançada de enfermagem para cuidar de mães de crianças com transtorno do espectro autista no programa CACTO^*^

**DOI:** 10.1590/1518-8345.8152.4883

**Authors:** Paulo Roberto Lima Falcão do Vale, Evany Caroline de Souza Cerqueira, Deisy Vital de Melo, Ana Paula Dezoti, Verônica de Azevedo Mazza, Rosely Cabral de Carvalho, Evanilda Souza de Santana Carvalho

**Affiliations:** 1 Universidade Federal do Recôncavo da Bahia, Centro de Ciências da Saúde, Santo Antônio de Jesus, BA, Brasil.; 2 Universidade Estadual de Feira de Santana, Departamento de Saúde, Feira de Santana, BA, Brasil.; 3 Centro Universitário Autônomo do Brasil, Departamento de Enfermagem, Curitiba, PR, Brasil.; 4 Universidade Federal do Paraná, Curitiba, PR, Brasil.; 5 Scholarship holder at the Conselho Nacional de Desenvolvimento Científico e Tecnológico (CNPq), Brasil.

**Keywords:** Prática Avançada de Enfermagem, Transtorno do Espectro Autista, Cuidadores, Enfermagem de Atenção Primária, Teoria de Enfermagem, Enfermagem Baseada em Evidências.

## Abstract

**Objetivo:**

descrever as competências da prática avançada de enfermagem para o cuidado de mães participantes do CACTO: programa de cuidado unitário às mães de crianças com transtorno do espectro autista e/ou deficiência.

**Métodos:**

estudo qualitativo fundamentado na Ciência do Cuidado Unitário, realizado com 39 mães de crianças com transtorno do espectro autista. Os dados foram coletados dos prontuários, diário de campo, atas de reuniões e do perfil do CACTO nas redes sociais. A análise temática reflexiva dedutiva foi aplicada como técnica de análise dos dados.

**Resultados:**

os resultados revelados ampliam o escopo de competências que podem ser desempenhadas pelas enfermeiras na atenção primária à saúde, por exemplo: compreende necessidades de saúde, cria novos modos de cuidar, reconhece dilemas éticos, estabelece relação colaborativa, valoriza o papel social das mães, sugere cuidados com evidência científica recomendada, realiza pesquisa científica e atua com autonomia.

**Conclusão:**

as competências do enfermeiro atuante no CACTO distribuem-se em sete dimensões, a saber: gestão do cuidado, ética, colaboração interprofissional, promoção da saúde e prevenção de riscos, enfermagem baseada em evidências, pesquisa e liderança.

## Destaques

**(1)** Competências do enfermeiro atuante no CACTO podem ampliar o escopo de prática na APS. **(2)** Criação e invenção de novos modos de cuidar podem elevar a resolutividade da APS. **(3)** Enfermeiros da APS devem buscar novas soluções para necessidades de saúde das mães. **(4)** Protagonismo dos usuários é fundamental para implementação de tecnologias de cuidado. **(5)** Enfermeiros da APS devem investir na subjetivação e capacidade de abstração.

## Introdução

A prevalência do Transtorno do Espectro Autista (TEA) tem aumentado de forma expressiva; estima-se que cerca de 1 a cada 31 crianças apresentam TEA no mundo^1^. No Brasil, aproximadamente dois milhões de pessoas convivem com o transtorno^2^. As mães assumem o papel principal na percepção das deficiências de seus filhos, gerenciamento do comportamento da criança, busca de terapias adequadas e construção do itinerário terapêutico necessário para desenvolvimento do seu filho^3^. Nesse cenário, as mães acessam com frequência os estabelecimentos de saúde de média densidade tecnológica^4^, por exemplo, os Centros Especializados em Reabilitação (CER), havendo poucas iniciativas de cuidado direcionadas às mães na Atenção Primária à Saúde (APS)^3^.

Dada a sobrecarga de cuidado, níveis elevados de ansiedade e depressão, baixa qualidade de vida e qualidade do sono, além da baixa adesão ao autocuidado e interrupção dos seus projetos de vida em busca da autonomia da criança^3^, a mãe convive com fatores de risco que a colocam em situação de vulnerabilidade. Devido às necessidades de saúde e carência de cuidados, cabe à APS aplicar cuidados favoráveis à promoção da saúde e emancipação da mãe, assumir medidas para enfrentamento da heteronormatividade hegemônica e reordenar a Rede de Atenção à Saúde (RAS) em linha de cuidado inclusiva para a díade mãe-criança. 

Ancorados nos atributos de vínculo, responsabilização sanitária, longitudinalidade e capilaridade, advoga-se por cuidados específicos às mães implementados na APS. Portanto, torna-se essencial o desenvolvimento de programas que atendam às necessidades de saúde das mães. Nesse sentido, o CACTO: Programa de Cuidado às Mães de Crianças com Síndrome Congênita do Zika^5^ foi desenvolvido e posteriormente aplicado para cuidar de mães de crianças com TEA^6^.

O CACTO, fundamentado na Ciência do Cuidado Unitário (CCU) proposta pela enfermeira Jean Watson^7^, viabiliza cuidados inovadores com abordagem humanitária que contrasta com práticas excludentes e individualistas, promovendo uma assistência mais integral, equitativa e emancipatória^6^. Atua sobre as repercussões de saúde vivenciadas pelas mães mediante conjunto de cuidados específicos para promover o bem-estar, reduzir vulnerabilidades, potencializar a existência humana e restauração (*healing*)^5^.

Lacunas de conhecimento, prática descontextualizada e repetitiva resultam em cuidados de enfermagem pouco eficazes, por vezes inadequados às necessidades das pessoas em situação de vulnerabilidade^8^. Nesse sentido, a Prática Avançada em Enfermagem (PAE) emerge como importante constructo que busca garantir a ação da Enfermeira, qualificando, principalmente, sua prática clínica na APS^9^.

A PAE corresponde à atuação da enfermeira a partir de conhecimentos especializados, habilidades complexas de tomada de decisão e competências clínicas ampliadas que lhe conferem melhor autonomia profissional, singularidades aos aspectos culturais, regulatórios e às necessidades específicas das populações atendidas^10^. O Enfermeiro de Prática Avançada (EPA) amplia seu escopo clínico e assume papéis estratégicos de liderança, gestão do cuidado, educação e pesquisa, articulando essas dimensões para qualificar a assistência com foco em segurança, efetividade, inovação e promoção da saúde^10^.

Na prática clínica, o EPA busca respeitar as singularidades da pessoa, família e comunidade, atuando como agente transformador no serviço de saúde. Sua formação acadêmica é avançada e contínua, o que sustenta um raciocínio clínico aprimorado, o uso crítico das melhores evidências científicas e o compromisso ético com a prevenção de riscos, a colaboração interprofissional, a gestão do cuidado, a autonomia profissional, a liderança e o domínio tecnológico, promovendo a evolução constante da enfermagem^10^, competências necessárias para a singularidade e qualidade do cuidado na APS, bem como elevação das taxas de resolutividade.

No cuidado às famílias de crianças com TEA, a enfermeira tem oferecido apoio emocional, orientado práticas para atenuação da sobrecarga do cuidado, articulado intervenções interprofissionais^11^, identificado sinais precoces do TEA e coordenado o itinerário terapêutico^12^. Contudo, são escassas evidências da PAE para mães de crianças com TEA e/ou deficiência.

O processo de implementação das PAEs deve ser guiado por evidências científicas que subsidiem a prática do EPA, como os conjuntos de competências organizados em domínios, a saber: gestão do cuidado, ética, colaboração interprofissional, promoção e prevenção, enfermagem baseada em evidências, pesquisa e liderança, diretamente alinhados com as diretrizes, subsidiando a prática do EPA^9^. Por isso se impõe o desafio da formação, qualificação e desenvolvimento de competências das enfermeiras para o cuidado longitudinal de pessoas em situação de vulnerabilidade, assim como a valorização dos usuários enquanto protagonistas de uma tecnologia culturalmente apropriada como o CACTO. 

Dada a necessidade de modelos de cuidados inovadores implementados pela Enfermagem na APS, bem como a necessidade de regulamentar as competências para formação do EPA no Brasil, pretende-se responder à seguinte questão de pesquisa: Quais as competências da EPA aplicadas para o cuidado de mães de crianças com transtorno do espectro autista participantes do programa CACTO? O objetivo deste estudo é descrever as competências da prática avançada de enfermagem para o cuidado de mães participantes do Programa.

## Métodos

### Tipo do estudo

Estudo qualitativo a partir da implementação do programa CACTO fundamentado na Ciência do Cuidado Unitário^7^ enquanto referencial teórico. Este estudo foi redigido em atendimento às diretrizes do *Consolidated Criteria for Reporting Qualitative Research* (COREQ).

### Local do estudo e período da coleta de dados

Este estudo foi desenvolvido no âmbito do programa CACTO, o qual se iniciou em outubro de 2023 e segue em funcionamento no instituto TEAbraco SAJ até o momento. A coleta de dados para fins deste manuscrito foi realizada entre outubro de 2023 e janeiro de 2025.

O instituto é uma organização não governamental dirigida por mães de crianças com TEA, mantida por meio de doações e parcerias voluntárias, que oferece atendimento de fisioterapia, psicologia, fonoaudiologia, neuropsicopedagogia, musicoterapia e assistência social, apenas para as crianças. O acesso aos serviços terapêuticos ocorre via pagamento por desembolso direto das famílias; em casos específicos, após análise social, algumas famílias são beneficiadas com acesso gratuito aos serviços. Aproximadamente 260 famílias estão associadas ao instituto, localizado no município de Santo Antônio de Jesus (BA), com população residente de 103.055 pessoas, índice de desenvolvimento humano municipal de 0.700 e produto interno bruto de R$ 23.746,79.

### Referencial teórico

Em 1979, Jean Watson colocou em análise o cuidado humano, preocupada com o modo de interação entre a enfermeira e a pessoa a ser cuidada, que se distanciava progressivamente pela inserção de tecnologias duras, colocando em plano secundário o cuidado interacional. Emergiu então a Teoria do Cuidado Humano e os *Carative Factors* enquanto primeiro paradigma, para enfatizar o momento do cuidar enquanto evento-fim e não um evento-meio para se alcançar a cura^7^. 

A CCU é o constructo teórico atual da Watson mediante o paradigma Unitário-Transformativo, considerando que todos os seres estão interligados pela energia cósmica universal, uma fonte de vida que é o amor, o ponto de partida da ontologia do ser. A interação com cada pessoa requer a elevação da consciência da Enfermeira, presença genuína, abertura para dar e receber, aplicação dos valores morais e éticos, uso de habilidades, atitudes e conhecimentos da enfermagem enquanto ciência, o que favorece a transcendência^7^.

Buscando o letramento da Enfermagem sobre a CCU, Watson desenvolve o *Veritas*, representado por uma palavra vibrante, que emana energia positiva, vida. O cuidado *Caritas-Veritas* se sustenta no amor enquanto fonte de vida, na evolução da consciência e intencionalidade de cuidar para alcance do *healing* (*Caritas*), atribuindo a cada *Caritas* o seu componente moral e valor ético aplicado durante a prática de cuidar (*práxis*)^7^.

Os encontros de cuidado desenvolvidos no programa CACTO fundamentam-se na CCU^7^, respeitando os dez elementos do processo clínico *Caritas*-*Veritas* (PCC-V), valorizando a narrativa livre da mãe, evitando cerceamento da fala e conduzindo o pensamento para compreensão de si e planejamento de práticas de autocuidado. Os dez elementos do PCC-V são: Amor-Gentileza; Fé-Esperança; Eu-Transpessoal; Nutrir-Relacionar; Perdoar; Autocriar; Aprender; Ambiente *Caritas*; Humanidade; Infinidade^7^. 

No CACTO, o encontro de cuidado entre enfermeiro e mãe ocorre em seis passos, quando é possível aplicar cada elemento do PCC-V conforme Figura 1:

**Figura 1:** Seis passos para realização do encontro de cuidado fundamentado na Ciência do Cuidado Unitário

*PCC-V = Processo Clínico *Caritas-Veritas*

### Participantes

Autodeclarar-se mãe de criança com TEA foi o único critério de inclusão deste estudo. Por se tratar de um grupo de mulheres com necessidades pouco abordadas nos serviços de saúde, entender que o CACTO pode beneficiar quantidade significativa das mães e considerar que não havia variável que tornasse a amostra heterogênea, não foram definidos critérios de exclusão.

Foram convidadas 44 mães que frequentavam o Instituto TEAbraco SAJ nos turnos semanais de funcionamento do CACTO. Houve três recusas justificadas pela indisponibilidade de tempo, uma por estar em tratamento quimioterápico e outra por desacordo com o cônjuge, constituindo amostra final de 39 participantes, identificadas por códigos como M1, M2, M3..., M51.

### Instrumentos de coleta de dados

As fontes de dados foram os prontuários, preenchidos após o encontro de cuidado, diário de campo (organizado em ficha com as seguintes seções: descrição do fato, inferência, o que fazer?) e atas de reuniões, documentos elaborados pelo enfermeiro e armazenados no Google *Drive*, além de dados extraídos do perfil do CACTO no Instagram (@cacto.ufrb). Os encontros de cuidado foram audiogravados, transcritos e importados para prontuários individuais. O prontuário foi elaborado em atendimento à CCU, ao modelo de Registro Clínico Orientado por Problemas, método Subjetivo, Objetivo, Avaliação, Plano e à Classificação Internacional da Atenção Primária, edição 2 (CIAP-2). São apêndices do prontuário a ficha de anotações, acordos, plano de cuidados e intervenções; ficha de referência e contrarreferência e planilha de longitudinalidade do cuidado.

O prontuário encontra-se estruturado em dez seções, a saber: identificação sociodemográfica e econômica da mãe e criança; quem é você? história da vida atual; antecedentes pessoais; antecedentes familiares; história do TEA na criança; suporte social e relação com a criança; autocuidado e hábitos de vida; aspectos familiares e sociais (genograma e ecomapa); comentários finais e reflexões.

### Coleta de dados

Após autorização da direção do instituto, o enfermeiro do CACTO realizou primeiro diálogo com as mães, de modo individual, no espaço da recepção da sede do instituto, quando o enfermeiro realizou o convite para integrar o programa. As 39 mães foram convidadas a conhecer o espaço físico do CACTO, localizado na sede do instituto. A seguir, procedeu-se à leitura e assinatura do termo de consentimento livre e esclarecido e do termo de autorização do uso de imagens e sons. As mães foram informadas que os dados produzidos a partir dos encontros de cuidado seriam utilizados para fins de pesquisa.

O enfermeiro atuante no CACTO tem mais de 11 anos de formação em bacharel em Enfermagem e de especialista em Saúde da Família, modalidade residência multiprofissional em saúde. É especialista em Gestão em Saúde, mestre e doutor em Saúde Coletiva, com formação em diferentes práticas integrativas e complementares. Foi bolsista de iniciação científica em estudos com famílias durante três anos da graduação. Ao longo de toda a carreira profissional, atuou na Estratégia de Saúde da família (ESF) e como docente, ministrando componentes curriculares afins da atenção primária e saúde coletiva, e desenvolveu o CACTO durante doutoramento em Saúde Coletiva (2018-2022).

A função do enfermeiro atuante no CACTO inclui: organizar agendamento dos encontros de cuidado; preparar ambiente de cuidado; realizar registro no prontuário; elaborar plano de cuidado; coordenar o cuidado por meio do projeto terapêutico singular; qualificar a equipe mediante sessões de educação permanente; e captar recursos para funcionamento do programa e cuidar das mães. 

Para assegurar a confiabilidade da extração dos dados, os autores se reuniram previamente para compreender os domínios e competências do EPA; em seguida, foi elaborada uma matriz com os sete domínios do EPA em linhas e as quatro fontes de dados da pesquisa dispostas em coluna. Após sanar dúvidas epistemológicas e metodológicas, o primeiro e segundo autores preencheram, simultaneamente, a matriz em *software* Microsoft Office Word 2019, entre janeiro e fevereiro 2025. A fim de alcançar amplo escopo de competências identificadas, foram incluídos todos os dados alinhados com os domínios do EPA, por isso não houve divergência entre os autores durante o preenchimento da matriz.

### Análise de dados

A análise temática (AT) reflexiva do tipo dedutiva^13^ foi explorada a partir dos sete domínios de competências do EPA escolhido como lente de análise, a saber: gestão do cuidado, ética, colaboração interprofissional, promoção e prevenção, enfermagem baseada em evidências, pesquisa e liderança^9^. A primeira etapa consistiu no aprofundamento do corpo teórico, realizado antes da extração dos dados, através de leitura exaustiva, reuniões e sessões científicas sobre os domínios do EPA^9^. Seguiu-se com as seis fases da AT: familiarizando-se com os dados; codificação, quando os autores buscaram por códigos que atendessem aos domínios de competências, devido à grande quantidade de dados, uma segunda rodada de codificação foi necessária, resultando em 68 códigos; na fase gerando temas iniciais, a matriz de extração e análise dos dados com os 68 códigos foi disponibilizada no Google *Drive* para contribuição de todos os autores. Após sete dias, os autores se reuniram para emitir sugestões sobre os códigos e agrupá-los por semelhança, contextualização ou tempo cronológico, resultando em 38 temas, ou seja, 38 competências do enfermeiro. Os conflitos existentes foram resolvidos por consenso entre todos os autores. Na quarta fase, o primeiro e segundo autores retornaram ao banco de dados a fim de identificar dados convergentes e divergentes para consolidação das temáticas. Nenhum acréscimo ou retirada de dados foi realizada nessa fase. Na fase de refinar, definir e nomear temas, as competências foram reescritas, colocadas em reunião de discussão e reflexão com participação de todos os autores a fim de levantar possíveis inferências; por fim, a última fase consistiu na redação final.

### Considerações éticas

O estudo foi aprovado em Comitê de Ética em Pesquisa com Seres Humanos da Universidade Federal do Recôncavo da Bahia com o Certificado de Apresentação de Apreciação Ética n.º 65573422.5.1001.0056, parecer número 5.866.634.

## Resultados

Participaram 39 mães que se concentram de 36 a 40 anos (33,3%) e de 41 a 45 anos (20,5%), sendo 16 (41%) casadas e declaradas 19 (48,7%) pretas e 18 (46,1%) pardas. Vinte participantes (51,3%) concluíram o ensino médio, 15 (38,5%) são donas de casa, 16 (41%) se declararam sem renda e 14 (36%) têm renda entre R$ 600,00 e R$ 1.518,99. A idade das crianças com TEA declarada pelas mães foi predominantemente de 4 a 5 anos, distribuídas em 14 crianças (36,9%) e 11 (28,2%) entre 6 e 12 anos completos.

As competências do enfermeiro aplicadas à qualidade do cuidado de mães de crianças com TEA no CACTO e categorizadas por domínios estão descritas na Figura 2.

**Figura 2:** Domínios da EPA*, competências do Enfermeiro atuante no programa CACTO e evidências empíricas

Domínios	Competências	Evidências empíricas
**Gestão do cuidado**	**Enfoque do cuidado** Compreende as principais necessidades de saúde das mães.	Prescrição do acordo: Escrever uma carta contando o que a M12 de hoje escreveria para a M12 quando descobriu a gestação?
	Aplica a CCU^†^.	
	**Avaliação e diagnóstico** Explora o diálogo de modo amplo e exaustivo.	Registro em prontuário: *As pessoas falam: “ser mãe não é uma tarefa fácil”. Eu sabia que não era, eu não sabia que eu ia ficar sozinha, [...] no deserto, somente com meu filho, porque todas as portas estão fechadas* (M11).
	Realiza anamnese investigativa e aprofundada considerando a historicidade, traumas e experiências passadas significativas.	
	Utiliza gravadores de áudio para registro fiel dos dados e construção de banco de dados.	
	Aplica instrumentos de aferição dos níveis de ansiedade, depressão, esperança, resiliência, fadiga, sobrecarga do cuidado e qualidade de vida.	
	Padroniza o diagnóstico a partir da CIAP-2^‡^, recomendado pelo Ministério da Saúde do Brasil.	Registro em prontuário (principais problemas): Z12 - problema de relacionamento com o parceiro conjugal; P01 - sensação de ansiedade/nervosismo/tensão; T07 - aumento de peso.
	**Prestação do cuidado** Elabora e reavalia PTS^§^ junto com a mãe.	Registro em prontuário: *Fiz o acordo. Funcionou. Eu fui menos pra casa do meu pai, saí mais sozinha, fiz programas com meus filhos. Lá em meu pai fico muito sobrecarregada* (M56).
	Realiza apoio emocional, escuta ativa e toque/abraço terapêutico.	
	Orienta quanto à fisiologia, comportamentos esperados e convívio com a criança com TEA^ǁ^.	
	Direciona as mães quanto aos fluxos da RAS^¶^.	
	Realiza práticas integrativas e complementares.	Postagem publicada no perfil do CACTO. Disponível via link: https://www.instagram.com/p/C6MZ1wFAEr8/?igsh=MXVheHc3eHZtc2p1Yw==
	Prescreve uso de ervas e chás, bem como recomenda a manutenção de medicamentos alopáticos comumente utilizados.	Prescrito acordo para M51: Consumir chá de mulungu (3g/300ml de água). Uso: quatro xícaras de 300 ml por dia, feito por método de decocção (ferver com tampa aberta) (M51).
	Implementa os acordos de cuidado, como: “apreciar o silêncio”, “valorizar a presença do eu” e “realizar exercícios de contemplação”.	Registro do prontuário: *Eu fiz o acordo, dormi melhor, preparei o ambiente, tirei o celular, deu certo* (M01).
	Prescreve as modalidades de cuidado previstas no CACTO.	Prescrito acordo para M23: Praticar a cartilha cuidando da minha mente durante 21 dias. Lembrar de acessar os *podcasts* disponíveis.
	Cria novos modos de cuidar a partir das necessidades das mães.	Ficha de intervenção: “Desenhe algo que representa a mulher ideal” (Figura 3).
**Ética**	Constrói ambiente terapêutico que preserva sigilo, privacidade e conforto.	Postagens publicadas no destaque “Ambiência” no perfil do CACTO. Disponível via link: https://www.instagram.com/s/aGlnaGxpZ2h0OjE3OTMwOTM1ODMyOTY5NzUx?story_media_id=3256359463652818529_61260366393&igsh=b2VuYXgyN3p3N2wz
	Integra a presença da criança no momento de cuidado.	
	Respeita os princípios éticos da bioética, direitos humanos e ética profissional.	Registro no prontuário: *Eu não queria voltar para o interior, mas o pai dele quis, aí eu vim. Eu sei que ele me trai, tem outras, mas não quero que meu filho seja criado sem pai* (M41).
	Reconhece dilemas éticos das mães, como separar-se do genitor e “criar o filho sem pai” ou suportar situações de violência e infidelidade.	
**Colaboração interprofissional**	Realiza discussão de casos com profissionais da equipe multidisciplinar.	Ata de reunião: Ao décimo quarto dia do mês de novembro de 2024 reuniu-se a equipe de saúde do instituto para discussão da evolução do processo terapêutico de (nome omitido) e sua mãe M41 [...]
	Estabelece relação colaborativa com direção do instituto.	
	Preenche ficha de referência para compartilhamento de casos com profissionais da RAS^¶^.	
**Promoção e prevenção**	Estimula as mães a reconhecerem suas qualidades, valorizarem a si e se priorizarem.	Prescrito acordo para M44: Quais as qualidades e desafios que eu tenho?
	Valoriza o papel social exercido pelas mulheres.	
	Incentiva a adoção de hábitos de alimentação saudável, diminuição do etilismo e superação do sedentarismo.	Prescrito acordo para M10: Substituir um dos lanches por fruta da sua preferência. Ingerir junto com fibras: linhaça, chia, farinha de aveia ou outros.
	Desenvolve materiais informativos sobre enfrentamento ao capacitismo e divulga-os em redes sociais.	
**Enfermagem baseada em evidências**	Constrói e utiliza modalidades de cuidado sustentadas em evidências.	Desenvolvimento da modalidade de cuidado “cuidando do meu sono”^5^, fundamentado nas diretrizes da Associação Brasileira do Sono. Prescrito acordo para M1: iniciar o passo a passo cuidando do meu sono.
	Propõe metas no PTS^§^ fundamentadas por evidências clínicas.	
	Aplica protocolos do Ministério da Saúde do Brasil.	
**Pesquisa**	Desenvolve e orienta pesquisas para aferir fenômenos vivenciados pelas mães.	Postagens publicadas no perfil do CACTO. Disponível via link: https://www.instagram.com/p/C6KbRzVgN0F/?igsh=Znk5ejJoNXVtbTQ=
	Desenvolve projeto de pesquisa para expansão do CACTO a partir de edital de fomento do Ministério da Saúde do Brasil.	
	Conduz sessões científicas com os grupos de pesquisa parceiros.	
**Liderança**	Atua de maneira autônoma e com autoridade.	Registro no prontuário: consta na planilha de longitudinalidade a periodicidade e agendamento das consultas de cada mãe.
	Define periodicidade das consultas baseadas nas necessidades de cada mãe.	
	Agenda consulta da mãe concomitante à consulta da criança.	
	Promove estratégias de cuidado coletivo por meio do Grupo Vivências e da técnica do Grupo Focal.	Diário de Campo do Grupo Vivências realizado no dia 14/08/24: Mães compartilham experiências sobre o que fazer quando alguém criticar seu filho.
	Articula com a Residência em Medicina de Família e Comunidade e professores do curso de bacharelado em Enfermagem e Medicina.	Registro no prontuário: Ficha de referência preenchida e enviada para a médica de referência.

*EPA = Enfermagem de Prática Avançada; ^†^CCU = Ciência do Cuidado Unitário; ^‡^CIAP-2 = Classificação Internacional de Assistência Primária; ^§^PTS = Projeto Terapêutico Singular; ^||^ TEA = Transtorno do Espectro Autista; ^¶^RAS = Rede de Atenção à Saúde

**Figura 3:** Desenho elaborado por M45 após intervenção criada pelo enfermeiro e estimulada pela frase: “Desenhe algo que representa a mulher ideal”

## Discussão 

Os resultados revelados neste estudo ampliam o escopo de competências que podem ser desempenhadas pelos enfermeiros na APS^14^ e oferecem evidências científicas que respaldam as práticas de cuidar em enfermagem direcionadas para o bem-estar da mãe, com repercussões positivas às crianças com TEA e demais membros da família. Nesse cenário, gestores e implementadores de políticas públicas podem optar pela qualificação de enfermeiros da APS para desenvolvimento e implementação de linhas de cuidado a fim de elevar as taxas de acessibilidade dos sistemas universais de saúde, resolutividade da APS, redução das iniquidades, melhora da qualidade de vida e diminuição dos custos^15^.

Muitos países carecem de programas de cuidado estruturados, capazes de integrar diagnóstico precoce, intervenções multidisciplinares contínuas e suporte longitudinal às famílias e pessoas com TEA^16^. Esse panorama reforça a necessidade de programas específicos e coordenados por enfermeiros, considerando que a maioria dos modelos existentes concentra-se apenas na etapa diagnóstica, sem garantir seguimento adequado ou apoio às demandas emocionais e socioeducativas do núcleo familiar^17^.

Até o momento de escrita deste artigo, não se observou existência de programas ou linha de cuidado dirigidos para mães de crianças com TEA, tampouco PAE fundamentadas em teorias de enfermagem^18^, como a CCU. Portanto, a gestão do cuidado no CACTO é realizada pelo enfermeiro coordenador do programa, que fomenta o protagonismo das mães, considerando o enfoque do cuidado, avaliação e diagnóstico e prestação do cuidado^9^.

O primeiro momento de desenvolvimento do CACTO denomina-se “compreensão”^5^; trata-se de compreender as necessidades de saúde das mães, logo, importa que o enfermeiro acesse ampla variedade de literatura científica com níveis de evidência e grau de recomendações elevados, além de colher informações essenciais através das visitas domiciliárias. Por isso, antes da implementação do CACTO, considerando o subdomínio enfoque do cuidado, sabia-se que as mães necessitavam de acesso a direitos de proteção social, bens e serviços como moradia e saneamento básico, segurança alimentar, trabalho e escolaridade; serviços qualificados e cuidado multiprofissional empático; acolhimento, fortalecimento de vínculos e aceitação; responsabilização do genitor e compartilhamento das responsabilidades diárias e cuidado de si^5^.

O enfoque do cuidado no CACTO fundamenta-se na CCU, buscando o *healing* (restauração) do ser vivo a partir do equilíbrio entre as dimensões corpo-mente-alma que se dá durante e após o cuidado transpessoal^7^. O enfermeiro atuante no CACTO desenvolveu período de intercâmbio durante seu doutoramento, com objetivo de dominar a CCU, experiência que contribuiu para planejar a aplicabilidade dos atributos da APS durante implementação do CACTO, como territorialização, vínculo, participação social, responsabilização sanitária e protagonismo dos usuários^18^.

O diálogo amplo e exaustivo foi a técnica explorada durante a anamnese para melhor compreensão dos riscos e problemas determinantes do processo de adoecimento das mães. A concepção das pessoas enquanto sujeito histórico, constituído das experiências vivenciadas que moldam suas crenças e comportamentos^19^, justifica a necessidade de realização de anamnese investigativa e aprofundada, com coleta de dados desde o próprio nascimento até o momento presente, identificando traumas, tristezas, prazeres e alegrias vivenciados. Nesse momento é oportuna a valorização do processo de trabalho do Agente Comunitário de Saúde de referência da mãe e sua contribuição na elaboração dos PTS dos usuários^20^.

A avaliação e diagnóstico na PAE é um processo contínuo que envolve a coleta e análise de dados objetivos e subjetivos. Isso permite revisar diagnósticos, listas de problemas, planos de cuidado, intervenções e estratégias a partir do protagonismo do paciente e sua rede de apoio^18^. No processo diagnóstico, o EPA deve utilizar dados da avaliação clínica atribuídos aos sistemas padronizados de classificação clínica^21^ como o CIAP-2 explorado no CACTO. 

O EPA interessado em aplicar a CCU^7^ deve exercer habilidades singulares como capacidade de abstração, compreensão da totalidade constituinte do Ser humano sagrado, reconhecimento do dito e não dito, das expressões e silenciamentos, controle dos impulsos profissionais e pessoais que ceifam o pensamento das pessoas em cuidado, criatividade para criação das intervenções (Figura 1) e acordos, bem como agilidade para implementá-las em momentos oportunos.

Os dilemas éticos vivenciados pelo enfermeiro no CACTO reservam complexidade que urge processos decisórios delicados e prudentes a partir das necessidades de saúde das mães e suas prioridades. São experiências entre separar-se do cônjuge e pôr fim às situações de violência ou manter-se na relação conjugal por dependência financeira; ou ainda, retomar os objetivos profissionais e sentir-se culpada por terceirizar o cuidado da criança ou continuar cuidando integralmente da criança e conviver com dificuldades financeiras. As decisões éticas do enfermeiro baseiam-se em legislações profissionais, protocolos institucionais e na Declaração Universal sobre Bioética e Direitos Humanos, além do princípio da sensibilidade moral, que permite reconhecer a dimensão ética dos problemas e envolver o paciente no processo decisório^22^.

A colaboração interprofissional fomenta uma cultura de respeito mútuo, confiança e reconhecimento pelas contribuições de cada membro da equipe, criando um ambiente propício à inovação, criatividade e excelência no atendimento. Dessa forma, potencializa a partilha de saberes, aprimora o cuidado e a tomada de decisão, aumentando a segurança do ser cuidado e otimizando recursos^23^. O cuidado às mães exige cumprimento das funções da APS como coordenadora do cuidado e ordenadora da RAS para otimização do tempo de resposta, abreviação dos itinerários terapêuticos para si e para as crianças e aumento da resolutividade a partir das possibilidades de matriciamento junto à equipe multidisciplinar na APS (eMulti) e aos terapeutas das crianças que compõem a RAS. O princípio da intersetorialidade também deve ser coordenado pela equipe da APS para atendimento das necessidades de saúde das mães junto ao Centro de Referência de Assistência Social (CRAS) e escolas/creches frequentadas por seus filhos.

As necessidades das mães manifestam-se enquanto problemas emocionais, comportamentais, psicossociais e clínico-biológicos que podem ser compreendidos enquanto domínios corpo, mente e alma^17^. A avaliação e diagnóstico indicam que o enfermeiro atuante no CACTO deve explorar habilidades no gerenciamento do cuidado das condições crônicas; portanto, as ações de prevenção de riscos e agravos e promoção da saúde são prioritárias. As evidências empíricas relacionadas ao cumprimento dos acordos pactuados no CACTO apontam para elevada satisfação das mães, assim como outros estudos incluídos em revisão sistemática indicam que a atuação do EPA impacta positivamente na qualidade de vida dos pacientes^24^.

O manejo das condições crônicas orienta-se pela valorização de si, reconhecimento das potencialidades e limites, conscientização do próprio processo saúde-doença, alinhamento das expectativas de cura, grau de autodeterminação, enfrentamento e motivação^25^. Os atributos e funções da ESF, como acessibilidade, vínculo, acolhimento, humanização, territorialização, responsabilização sanitária, coordenação e longitudinalidade do cuidado^26^ foram elementos aplicados pelo enfermeiro do CACTO e contribuíram para revelar demandas ocultas das mães, elevar a taxa de assiduidade às consultas, promover adesão aos acordos implementados e favorecer a valorização social do papel materno e autoestima.

A variedade de diagnósticos realizados e a característica singular e personalizada do CACTO exigiram a implementação de amplo escopo de cuidados, assim como a criação de novos cuidados baseados em evidências. O EPA deve desenvolver competências de capacidade investigativa para encontrar as melhores evidências científicas e elucidar questionamentos que surgem no campo de prática. As competências do domínio pesquisa contribuem para a inovação a partir do desenvolvimento de novas pesquisas, protocolos de promoção de saúde e implementação de novas tecnologias de cuidado na APS^9^.

Vale ainda salientar, a partir dessa experiência, a condição fundamental de protagonismo dos usuários para o desenvolvimento de tecnologias culturalmente apropriadas. A diversidade e amplo escopo das competências do enfermeiro do CACTO endossam a relevância dos cursos de nível *stricto sensu* para aperfeiçoar os produtos técnicos, incentivando o desenvolvimento de tecnologias sociais, aprofundamento epistemológico e aplicabilidade das teorias de enfermagem, bem como o gerenciamento de programas de cuidado, a Enfermagem enquanto ciência e a implementação da PAE na APS.

Os enfermeiros atuantes na APS necessitam apresentar características como comprometimento com a profissão, análise crítica e reflexiva, capacidade de abstração, habilidades educacionais e clínicas consolidadas pela formação qualificada em ensino e pesquisa. Tais aspectos permitiram atuar clinicamente em face da variedade de diagnósticos, prestar diversidades de cuidados, coordenar planos de cuidados, promover a longitudinalidade do cuidado, além de expressar autonomia e autoridade, qualidades esperadas pelo *Nurse Practitioner* ^14^ *.*

Os resultados indicam o fortalecimento da identidade profissional do enfermeiro do CACTO, ao potencializar o raciocínio clínico do enfermeiro e o papel de liderança na equipe interprofissional^27^ e proporcionar soluções para questões críticas ainda não respondidas a partir da implementação de programas, políticas ou práticas destinadas a produzir impactos de acesso e iniquidades para pessoas em situação de vulnerabilidade. As evidências desse estudo valorizam a qualidade da formação em enfermagem, apontam a relevância da APS e a necessidade da formação adicional em PAE na pós-graduação, com ressalvas para as especificidades regionais de cada país.

Achados de estudo do tipo revisão de escopo revelaram que a atuação do EPA na APS está associada a menos visitas de pacientes em pronto-socorro, menor incidência de internações ou reinternações hospitalares, menos consultas na APS, maior adesão aos planos de cuidado e menor custo com gastos relacionados a saúde^28^. A formação do EPA mediante curso de doutorado de prática de enfermagem com ênfase na APS é uma iniciativa em expansão ao redor do mundo^29^.

Etnografia realizada com enfermeiras da Suíça identificou funções do EPA na APS, a saber: definição das funções e funcionamento da equipe de saúde, resolução de conflitos interprofissionais, cuidado centrado na pessoa, liderança colaborativa e comunicação interprofissional^30^. No contexto internacional, o EPA tem contribuído para ampliar o acesso à APS, oferecer cuidados favoráveis ao envelhecimento ativo e manejar os cuidados às pessoas com doenças crônicas^31^. 

No Reino Unido, os EPA e enfermeiros de família que trabalham na comunidade desenvolvem competências semelhantes, com maior atuação do EPA na dimensão pesquisa, enquanto os enfermeiros de família atuam frequentemente nos domínios educação e liderança^32^. Contudo, diversos estudos convergem ao entendimento de que particularidades legais e as necessidades territoriais de cada país devem definir o escopo de atuação do EPA^28^ ^-^ ^30^.

Este estudo contribui para o avanço do conhecimento científico ao apresentar iniciativas inovadoras para cuidado a um público em amplo crescimento e com necessidades de saúde pouco exploradas nos serviços de saúde, as mães de crianças com TEA. Entende-se que as competências encontradas podem apoiar enfermeiras para um cuidado alinhado com as necessidades das mães. Os resultados permitem discutir as competências desempenhadas pelo enfermeiro do CACTO e aquelas aplicadas por enfermeiros da APS ao redor do mundo; portanto, próximos estudos podem examinar e avaliar a qualidade da assistência às mães de crianças com TEA e o processo de formação do Enfermeiro da APS, considerando as necessidades de saúde das mães.

Compreende-se enquanto limitação do estudo as especificidades do instituto TEAbraco SAJ fundamentadas nos princípios do associativismo, voluntarismo e solidariedade, que podem ter influenciado os aspectos motivacionais, processos decisórios e as relações intersubjetivas entre enfermeiro e mães. Devido a isso, as evidências encontradas devem ser aplicadas com ressalvas em outros espaços de cuidado da RAS.

## Conclusão

As competências do enfermeiro atuante no CACTO se organizam em sete dimensões, a saber: gestão do cuidado, ética, colaboração interprofissional, promoção e prevenção, enfermagem baseada em evidências, pesquisa e liderança. Entre as competências, destacam-se a criação e invenção de novos modos de cuidar, autonomia no cuidado das mães, prudência em dilemas éticos, capacidade investigativa e de pesquisa, bem como liderança e protagonismo para coordenação do cuidado e organização da RAS, a fim de ampliar o escopo de prática na APS e elevar a resolutividade. 

O enfermeiro atuante no CACTO desempenhou competências que podem ser aplicadas por enfermeiros na APS a partir de estratégias para ampliar o escopo de práticas da Enfermagem, como promover o autocuidado das mães, contribuir com o desenvolvimento psicossocial e cognitivo das crianças, responsabilizar genitores e agir politicamente a favor da inclusão social das famílias de crianças com TEA.

## Data Availability

Todos os dados gerados ou analisados durante este estudo estão incluídos neste artigo publicado.

# Translation: Práctica avanzada de enfermería para el cuidado de madres de niños con trastorno del espectro autista en el programa CACTO^*^

**DOI:** 10.1590/1518-8345.8152.4881

**Authors:** Paulo Roberto Lima Falcão do Vale, Evany Caroline de Souza Cerqueira, Deisy Vital de Melo, Ana Paula Dezoti, Verônica de Azevedo Mazza, Rosely Cabral de Carvalho, Evanilda Souza de Santana Carvalho

**Affiliations:** 1 Universidade Federal do Recôncavo da Bahia, Centro de Ciências da Saúde, Santo Antônio de Jesus, BA, Brasil.; 2 Universidade Estadual de Feira de Santana, Departamento de Saúde, Feira de Santana, BA, Brasil.; 3 Centro Universitário Autônomo do Brasil, Departamento de Enfermagem, Curitiba, PR, Brasil.; 4 Universidade Federal do Paraná, Curitiba, PR, Brasil.; 5 Scholarship holder at the Conselho Nacional de Desenvolvimento Científico e Tecnológico (CNPq), Brasil.

**Keywords:** Enfermería de Práctica Avanzada, Trastorno del Espectro Autista, Cuidadores, Enfermería de Atención Primaria, Teoría de Enfermería, Enfermería Basada en la Evidencia.

## Abstract

**Objetivo:**

describir las competencias de la práctica avanzada de enfermería para el cuidado de madres que participan en CACTO: un programa de atención unitaria para madres de niños con trastorno y/o discapacidad del espectro autista.

**Métodos:**

un estudio cualitativo basado en la Ciencia del Cuidado Unitario, realizado con 39 madres de niños con trastorno del espectro autista. Los datos se recopilaron a partir de registros médicos, diarios de campo, actas de reuniones y el perfil de CACTO en redes sociales. Se aplicó el análisis temático reflexivo deductivo como técnica de análisis de datos.

**Resultados:**

los resultados revelados amplían el alcance de competencias que pueden desempeñar los enfermeros en atención primaria, por ejemplo: comprenden las necesidades de salud, crean nuevas formas de cuidar, reconocen dilemas éticos, establecen relaciones de colaboración, valoran el papel social de las madres, sugieren cuidados con la evidencia científica recomendada, realizan investigaciones científicas y actúan con autonomía.

**Conclusión:**

las competencias de las enfermeras que trabajan en CACTO se distribuyen en siete dimensiones, a saber: gestión del cuidado, ética, colaboración interprofesional, promoción de la salud y prevención de riesgos, enfermería basada en la evidencia, investigación y liderazgo.

## Destacados

**(1)** Las competencias de la enfermera que trabaja en CACTO pueden ampliar el ámbito de práctica en la APS. **(2)** La creación e invención de nuevas formas de cuidar puede aumentar la capacidad de resolución de problemas de la APS. **(3)** Los enfermeros de la APS deben buscar nuevas soluciones para las necesidades de salud de las madres. **(4)** El protagonismo de los usuarios es fundamental para la implementación de tecnologías de atención. **(5)** Los enfermeros de la APS deben invertir en la capacidad de subjetivación y abstracción.

## Introducción

La prevalencia del Trastorno del Espectro Autista (TEA) ha aumentado significativamente; se estima que aproximadamente 1 de cada 31 niños tiene TEA en el mundo^1^. En Brasil, aproximadamente dos millones de personas viven con este trastorno^2^. Las madres asumen el papel principal en la percepción de las discapacidades de sus hijos, la gestión del comportamiento infantil, la búsqueda de terapias adecuadas y la elaboración del itinerario terapéutico necesario para el desarrollo de su hijo^3^. En este escenario, las madres acuden frecuentemente a centros de salud con densidad tecnológica media^4^, por ejemplo, los Centros Especializados de Rehabilitación (CER), y hay pocas iniciativas de atención dirigidas a madres en Atención Primaria de Salud (APS)^3^.

Dada la sobrecarga de cuidados, los altos niveles de ansiedad y depresión, la baja calidad de vida y de sueño, además de la baja adhesión al autocuidado y la interrupción de sus proyectos vitales en busca de la autonomía del niño^3^, la madre vive con factores de riesgo que la sitúan en una situación de vulnerabilidad. Debido a las necesidades sanitarias y la falta de atención, corresponde a la APS aplicar cuidados favorables a la promoción de la salud y la emancipación de la madre, tomar medidas para enfrentar la heteronormatividad hegemónica y reorganizar la Red de Atención de Salud (RAS) en una línea de atención inclusiva para la díada madre-hijo.

Basándonos en los atributos de vínculo, responsabilidad sanitaria, longitudinalidad y capilaridad, abogamos por la implementación de cuidados específicos para las madres en la APS. Por ello, es esencial desarrollar programas que respondan a las necesidades de salud de las madres. En este sentido, se desarrolló el CACTO: Programa de Atención a Madres de Niños con Síndrome de Zika Congénito^5^ y posteriormente se aplicó para el cuidado de madres de niños con TEA^6^.

El CACTO, basado en la Ciencia del Cuidado Unitario (CCU) propuesta por la enfermera Jean Watson^7^, permite una atención innovadora con un enfoque humanitario que contrasta con prácticas excluyentes e individualistas, promoviendo una atención más integral, equitativa y emancipadora^6^. Actúa sobre las repercusiones en la salud que experimentan las madres a través de un conjunto de cuidados específicos para promover el bienestar, reducir vulnerabilidades, mejorar la existencia humana y restauración curativa^5^.

Las lagunas de conocimiento, la práctica descontextualizada y la repetición resultan en una atención de enfermería ineficaz, a veces inadecuada para las necesidades de personas en situaciones vulnerables^8^. En este sentido, la Práctica Avanzada de Enfermería (PAE) surge como un constructo importante que busca garantizar la acción de las enfermeras, principalmente calificando su práctica clínica en la APS^9^.

La PAE corresponde al desempeño de la enfermera basado en conocimientos especializados, habilidades complejas para la toma de decisiones y competencias clínicas ampliadas que le otorgan una mayor autonomía profesional, singularidades en aspectos culturales y regulatorios y a las necesidades específicas de las poblaciones atendidas^10^. La Enfermera de Práctica Avanzada (EPA) amplía su ámbito clínico y asume roles estratégicos en liderazgo, gestión de cuidados, educación e investigación, articulando estas dimensiones para calificar la atención con un enfoque en la seguridad, la eficacia, la innovación y la promoción de la salud^10^.

En la práctica clínica, la EPA busca respetar las singularidades de la persona, la familia y la comunidad, actuando como un agente transformador en el servicio de salud. Su formación académica es avanzada y continua, lo que apoya un razonamiento clínico mejorado, el uso crítico de la mejor evidencia científica y el compromiso ético con la prevención de riesgos, la colaboración interprofesional, la gestión de cuidados, la autonomía profesional, el liderazgo y el dominio tecnológico, promoviendo la evolución constante de la enfermería^10^, competencias necesarias para la singularidad y calidad de la atención en la APS, así como un aumento en las tasas de resolución.

En el cuidado de familias de niños con TEA, las enfermeras han ofrecido apoyo emocional, han guiado prácticas para mitigar la carga de cuidados, han articulado intervenciones interprofesionales^11^, han identificado signos tempranos de TEA y han coordinado el itinerario terapéutico^12^. Sin embargo, hay poca evidencia de PAE para madres de niños con TEA y/o discapacidades.

El proceso de implementación de las PAE debe guiarse por la evidencia científica que respalde la práctica de la EPA, como los conjuntos de competencias organizados en dominios, a saber: gestión de la atención, ética, colaboración interprofesional, promoción y prevención, enfermería basada en evidencias, investigación y liderazgo, directamente alineadas con las directrices y apoyando la práctica de la EPA^9^. Por esta razón, se impone el desafío de formar, cualificar y desarrollar las habilidades de las enfermeras para la atención longitudinal de personas en situaciones vulnerables, así como la valoración de los usuarios como protagonistas de una tecnología culturalmente apropiada como el CACTO.

Dada la necesidad de modelos de atención innovadores implementados por la Enfermería en la APS, así como la necesidad de regular las competencias para la formación de la EPA en Brasil, con el proposito de responder a la siguiente pregunta de investigación: ¿Cuáles son las competencias de la EPA aplicadas al cuidado de madres de niños con trastorno del espectro autista que participan en el programa CACTO? El objetivo de este estudio es describir las competencias de la práctica avanzada de enfermería para el cuidado de las madres que participan en el Programa.

## Métodos

### Tipo do estudio

Este estudio cualitativo se basó en la implementación del programa CACTO basado en la Ciencia del Cuidado Unitario^7^ como marco teórico. Este estudio fue redactado en cumplimiento de las directrices de los Criterios Consolidados para la Comunicación de la Investigación Cualitativa (COREQ).

### Local del estudio y período de recopilación de datos

Este estudio se desarrolló dentro del marco del programa CACTO, que comenzó en octubre de 2023 y continúa operando en el instituto TEAbraco SAJ hasta la fecha. La recopilación de datos para los fines de este manuscrito se llevó a cabo entre octubre de 2023 y enero de 2025.

El instituto es una organización no gubernamental dirigida por madres de niños con TEA, mantenida mediante donaciones y colaboraciones voluntarias, que ofrece fisioterapia, psicología, logopedia, neuropsicopedagogía, musicoterapia y asistencia social únicamente para niños. El acceso a los servicios terapéuticos se realiza mediante pagos directos por parte de las familias; En casos concretos, tras el análisis social, algunas familias se benefician del acceso gratuito a los servicios. Aproximadamente 260 familias están asociadas al instituto, situado en el municipio de Santo Antônio de Jesus (BA), con una población residente de 103.055 personas, un índice municipal de desarrollo humano de 0.700 y un producto interior bruto de R$ 23.746,79.

### Referencial teórico

En 1979, Jean Watson analizó el cuidado humano, centrado en el modo de interacción entre la enfermera y la persona a atender, que se estaba distanciando progresivamente debido a la inserción de tecnologías duras, situando la atención interaccional en un plan secundario. La Teoría del Cuidado Humano y los Factores Carativos surgió como el primer paradigma para enfatizar el momento del cuidado como un evento final y no como un evento de medios para lograr una cura^7^.

La CCU es la construcción teórica actual de Watson a través del paradigma unitario-transformador, considerando que todos los seres están interconectados por energía cósmica universal, una fuente de vida que es el amor, el punto de partida de la ontología del ser. La interacción con cada persona requiere la elevación de la conciencia de la enfermera, una presencia genuina, apertura para dar y recibir, aplicación de valores morales y éticos, uso de habilidades, actitudes y conocimientos de la enfermería como ciencia, que favorece la trascendencia^7^.

Buscando la alfabetización de la enfermería sobre la CCU, Watson desarrolla *Veritas*, representado por una palabra vibrante, que emana energía positiva: vida. El cuidado de *Caritas* se basa en el amor como fuente de vida, en la evolución de la conciencia y la intencionalidad del cuidado para lograr la restauración curativa (*Caritas*), atribuyendo a cada *Caritas* su componente moral y su valor ético aplicado durante la práctica del cuidado (praxis)^7^.

Las reuniones de atención desarrolladas en el programa CACTO se basan en la CCU^7^, respetando los diez elementos del proceso clínico de *Caritas*-*Veritas* (PCC-V), valorando la narrativa libre de la madre, evitando la restricción del habla y conduciendo al pensamiento hacia la autocomprensión y planificación de prácticas de autocuidado. Los diez elementos del PCC-V son: Amor-Bondad; Fe-Esperanza; Yo transpersonal; Cuidado-relacionación; Perdonar; Créase a uno mismo; Aprende; *Caritas* Medio Ambiente; Humanidad; Infinito^7^.

En el CACTO, el encuentro de cuidado entre la enfermera y la madre ocurre en seis pasos, cuando es posible aplicar cada elemento del PCC-V como se muestra en la Figura 1:

**Figura 1:** Seis pasos para llevar a cabo la reunión de cuidados basándose en la Ciencia de la Atención Unitaria

*PCC-V: Proceso clínico de *Caritas*-*Veritas*

### Participantes

Autodeclararse madre de un niño con TEA fue el único criterio de inclusión en este estudio. Dado que se trata de un grupo de mujeres con necesidades poco atendidas en los servicios de salud, entendiendo que el CACTO puede beneficiar a un número significativo de madres y considerando que no existía ninguna variable que hiciera heterogénea a la muestra, no se definieron criterios de exclusión.

Se invitó a 44 madres que asistieron al Instituto TEAbraco SAJ en los turnos semanales de operación de CACTO. Hubo tres rechazos justificados por la falta de tiempo, uno porque estaban en tratamiento de quimioterapia y otro porque no estaban de acuerdo con el cónyuge, constituyendo una muestra final de 39 participantes, identificados por códigos como M1, M2, M3..., M51.

### Instrumentos de recogida de datos

Las fuentes de datos eran los historiales médicos, rellenados tras la reunión de cuidados, el diario de campo (organizado en un formulario con las siguientes secciones: descripción del hecho, inferencia,¿qué hacer?) y actas de reuniones, documentos preparados por la enfermera y almacenados en Google *Drive*, además de datos extraídos del perfil CACTO en Instagram (@cacto.ufrb). Las reuniones de atención fueron grabadas en audio, transcripciones e importadas en los registros médicos individuales. El historial médico fue elaborado en cumplimiento con la CCU, el modelo de Historial Clínico Orientado a Problemas, el Método Subjetivo, el Objetivo, la Evaluación, el Plan y la Clasificación Internacional de Atención Primaria, edición 2 (ICPC-2). Los apéndices del historial médico son el formulario de notas, acuerdos, plan de cuidados e intervenciones; Formulario de referencia y contrarreferencia y hoja de cálculo de longitudinalidad del cuidado.

El historial médico está estructurado en diez secciones, a saber: identificación sociodemográfica y económica de la madre y el hijo; ¿Quién eres? historia de la vida actual; historia personal; historia familiar; historia del TEA en niños; apoyo social y relación con el niño; el autocuidado y los hábitos de vida; aspectos familiares y sociales (genograma y ecomapa); Comentarios y reflexiones finales.

### Recogida de datos

Tras la autorización de la dirección del instituto, la enfermera de CACTO mantuvo el primer diálogo con las madres, de forma individual, en el espacio de recepción de la sede del instituto, cuando la enfermera las invitó a unirse al programa. Las 39 madres fueron invitadas a visitar el espacio físico de CACTO, ubicado en la sede del instituto. A continuación, se leyó y firmó el formulario de consentimiento libre e informado y el formulario de autorización para el uso de imágenes y sonidos. Se informó a las madres de que los datos obtenidos en las reuniones de cuidado se utilizarían con fines de investigación.

La enfermera que trabaja en el CACTO cuenta con más de 11 años de formación en una licenciatura en Enfermería y es especialista en Salud Familiar, modalidad de residencia multiprofesional en salud. Es especialista en Gestión de la Salud, con un máster y un doctorado en Salud Colectiva, con formación en diferentes prácticas integrativas y complementarias. Fue becaria de iniciación científica en estudios con familias durante tres años tras graduarse. A lo largo de su carrera profesional, trabajó en la Estrategia Salud de la Familia (ESF) y como profesora, impartiendo componentes curriculares relacionados con la atención primaria y la salud colectiva, y desarrolló el CACTO durante su doctorado en Salud Colectiva (2018-2022).

El papel de la enfermera que trabaja en el CACTO incluye: organizar la programación de reuniones de cuidados; preparar un entorno de cuidado; el registro en el historial médico; preparar un plan de cuidados; coordinar la atención a través del proyecto terapéutico singular; calificar al equipo mediante sesiones de formación permanente; y la recaudación de fondos para el funcionamiento del programa y el cuidado de las madres.

Para garantizar la fiabilidad de la extracción de datos, los autores se reunieron previamente para comprender los dominios y competencias de la EPA; luego, se elaboró una matriz con los siete dominios de la EPA en filas y las cuatro fuentes de datos de investigación organizadas en columnas. Tras resolver dudas epistemológicas y metodológicas, el primer y el segundo autor completaron simultáneamente la matriz en el *software* Microsoft Office Word 2019, entre enero y febrero de 2025. Para lograr un amplio alcance de competencias identificadas, se incluyeron todos los datos alineados con los dominios de la EPA, de modo que no hubo divergencia entre los autores durante la finalización de la matriz.

### Análisis de datos

Se exploró el análisis temático reflexivo (AT) del tipo deductivo^13^ basándose en los siete dominios de competencias de la EPA elegidos como lente de análisis, a saber: gestión del cuidado, ética, colaboración interprofesional, promoción y prevención, enfermería basada en la evidencia, investigación y liderazgo^9^. La primera etapa consistió en profundizar el cuerpo teórico, realizado antes de la extracción de datos, mediante lecturas exhaustivas, reuniones y sesiones científicas sobre los dominios de la EPA^9^. A esto le siguieron las seis fases de la AT: familiarizarse con los datos; la codificación, cuando los autores buscaban códigos que cumplieran con los dominios de competencia, debido a la gran cantidad de datos, era necesaria una segunda ronda de codificación, que resultaba en 68 códigos; en la generación de fases de los temas iniciales, la matriz de extracción y análisis de datos con los 68 códigos se puso a disposición en Google *Drive* para la contribución de todos los autores. Tras siete días, los autores se reunieron para hacer sugerencias sobre los códigos y agruparlos por similitud, contextualización o tiempo cronológico, resultando en 38 temas, es decir, 38 competencias de enfermeros. Los conflictos existentes se resolvieron por consenso entre todos los autores. En la cuarta fase, los primeros y segundos autores regresaron a la base de datos para identificar datos convergentes y divergentes para la consolidación de los temas. No se realizaron añadidos ni retiradas de datos en esta fase. En la fase de refinamiento, definición y nombramiento de temas, las competencias fueron reescritas, colocadas en una reunión de discusión y reflexión con la participación de todos los autores para plantear posibles inferencias; Finalmente, la última fase consistió en la redacción final.

### Consideraciones éticas

El estudio fue aprobado por el Comité de Ética en Investigación Humana de la Universidad Federal del Recôncavo da Bahia con el Certificado de Presentación de Apreciación Ética n.º 65573422.5.1001.0056, opinión número 5.866.634.

## Resultados

Los participantes fueron 39 madres de entre 36 y 40 años (33,3%) y 41 a 45 años (20,5%), 16 (41%) casadas y declaradas 19 (48,7%) negras y 18 (46,1%) marrones. Veinte participantes (51,3%) completaron la educación secundaria, 15 (38,5%) son amas de casa, 16 (41%) declararon no tener ingresos y 14 (36%) tienen ingresos entre R$ 600,00 y R$ 1.518,99. La edad de los niños con TEA declarada por las madres era predominantemente de 4 a 5 años, distribuida en 14 niños (36,9%) y 11 (28,2%) entre 6 y 12 años.

Las competencias de las enfermeras aplicadas a la calidad de la atención de madres de niños con TEA en el CACTO y categorizadas por dominios se describen en la Figura 2.

**Figura 2:** Dominios de la EPA*, competencias de la enfermera que trabaja en el programa CACTO y evidencias empíricas

Dominios	Competencias	Evidencias empíricas
**Gestión de la atención**	**Enfoque de atención** Comprende las principales necesidades de salud de las madres.	Prescripción del acuerdo: ¿Escribir una carta contando qué escribiría la M12 de hoy a M12 cuando descubriera el embarazo?
	Aplica la CCU^†^.	
	**Evaluación y diagnóstico** Explora el diálogo de forma amplia y exhaustiva.	Registro en el historial médico: *La gente dice: “ser madre no es una tarea fácil”. Sabía que no lo era, no sabía que iba a estar sola, [...] en el desierto, solo con mi hijo, porque todas las puertas están cerradas*(M11).
	Realiza análisis investigativos y en profundidad considerando la historicidad, los traumas y experiencias pasadas significativas.	
	Utiliza grabadoras de audio para el registro fiel de datos y la construcción de bases de datos.	
	Utiliza instrumentos para medir los niveles de ansiedad, depresión, esperanza, resiliencia, fatiga, sobrecarga de cuidados y calidad de vida.	
	Estandariza el diagnóstico basado en ICPC-2^‡^, recomendado por el Ministerio de Salud de Brasil.	Registro en el historial médico(principales problemas): Z12 - problema de relación con la pareja matrimonial; P01 - sensación de ansiedad/nervios/tensión; T07 - aumento de peso.
	**Prestación de cuidados** Elabora y reevalúa el PTS^§^ junto con la madre.	Registro en el historial médico: *Yo hice el acuerdo. Funcionó. Iba menos a casa de mi padre, salía más sola, hacía programas con mis hijos. Ahí en mi padre estoy muy abrumado* (M56).
	Realiza apoyo emocional, escucha activa y contacto terapéutico/abrazo.	
	Ofrece orientación sobre fisiología, comportamientos esperados y cómo convivir con el niño con TEA^ǁ^.	
	Dirige a las madres respecto a los flujos de RAS^¶^.	
	Lleva a cabo prácticas integrativas y complementarias.	Publicación publicada en el perfil de CACTO. Disponible a través de enlace: https://www.instagram.com/p/C6MZ1wFAEr8/?igsh=MXVheHc3eHZtc2p1Yw==
	Prescribe el uso de hierbas y tés, así como recomienda el mantenimiento de medicamentos alopáticos de uso común.	Acuerdo prescrito para M51: Consume té *mulungu* (3g/300ml de agua). Uso: cuatro tazas de 300 ml al día, elaboradas mediante método de decocción (hervir con tapa abierta) (M51).
	Implementa acuerdos de cuidado, tales como: “apreciar el silencio”, “valorar la presencia del yo” y “realizar ejercicios de contemplación”.	Registro en el historial médico: *Hice el acuerdo, dormí mejor, preparé el ambiente, saqué el móvil, funcionó* (M01).
	Prescribe las modalidades de atención previstas en el CACTO.	Acuerdo prescrito para M23: Practicar el manual cuidando mi mente durante 21 días. Recuerda acceder a los *podcasts* disponibles.
	Crea nuevas formas de cuidar basadas en las necesidades de las madres.	Hoja de intervención: “Dibuja algo que represente a la mujer ideal” (Figura 3).
**Ética**	Construye un entorno terapéutico que preserva la confidencialidad, la privacidad y el confort.	Las publicaciones publicadas en el “*Ambience*” destacan en el perfil de CACTO. Disponible a través del enlace: https://www.instagram.com/s/aGlnaGxpZ2h0OjE3OTMwOTM1ODMyOTY5NzUx?story_media_id=3256359463652818529_61260366393&igsh=b2VuYXgyN3p3N2wz
	Integra la presencia del niño en el momento del cuidado.	
	Respeta los principios éticos de bioética, derechos humanos y ética profesional.	Registro en el historial médico: *No quería volver al campo, pero su padre sí, así que fui. Sé que me traiciona, hay otros, pero no quiero que mi hijo se críe sin padre* (M41).
	Reconoce dilemas éticos de las madres, como separarse del progenitor y “criar al hijo sin padre” o soportar situaciones de violencia e infidelidad.	
**Colaboración interprofesional**	Lleva a cabo debates de casos con profesionales del equipo multidisciplinar.	Acta de la reunión: El día catorce de noviembre de 2024, el equipo sanitario del instituto se reunió para debatir la evolución del proceso terapéutico de (nombre no revelado) y su madre M41 [...]
	Establece una relación colaborativa con la dirección del instituto.	
	Rellena un formulario de referencia para compartir casos con profesionales de RAS^¶^.	
**Promoción y prevención**	Anima a las madres a reconocer sus cualidades, valorarse a sí mismas y priorizarse.	Acuerdo prescrito para M44: ¿Qué cualidades y desafíos tengo?
	Valora el papel social que desempeñan las mujeres.	
	Fomenta la adopción de hábitos alimentarios saludables, la reducción del alcoholismo y la superación de un estilo de vida sedentario.	Acuerdo prescrito para M10: Sustituye uno de los tentempiés por la fruta que elijas. Ingire junto con fibra: semillas de lino, chía, avena u otras.
	Desarrolla materiales informativos sobre la lucha contra el capacitismo y los difunde en redes sociales.	
**Enfermería basada en evidencia**	Construye y utiliza modalidades de atención basadas en la evidencia.	Desarrollo de la modalidad de cuidado “cuidando de mi sueño”^5^, basada en las directrices de la Asociación Brasileña del Sueño. Acuerdo prescrito para M1: empezar a cuidar mi sueño paso a paso.
	Propone objetivos en el PTS^§^ basándose en evidencia clínica.	
	Aplica protocolos del Ministerio de Salud de Brasil.	
**Investigación**	Desarrolla y guía investigaciones para evaluar fenómenos que experimentan las madres.	Publicaciones publicadas en el perfil de CACTO. Disponible a través del enlace: https://www.instagram.com/p/C6KbRzVgN0F/?igsh=Znk5ejJoNXVtbTQ=
	Desarrolla un proyecto de investigación para la ampliación de CACTO basado en un aviso de financiación del Ministerio de Salud de Brasil.	
	Realiza sesiones científicas con grupos de investigación asociados.	
**Liderazgo**	Actúa de forma autónoma y con autoridad.	Registro en el historial médico: la hoja de cálculo longitudinalidad contiene la periodicidad y la programación de las consultas de cada madre.
	Define la frecuencia de las consultas en función de las necesidades de cada madre.	
	Programa la cita de la madre de forma simultánea con la del niño.	
	Promueve estrategias de cuidado colectivo a través del Grupo de Experiencias y la técnica del Grupo Focal.	Diario de Campo del Grupo de Experiencias celebrado el 14/08/24: Las madres comparten experiencias sobre qué hacer cuando alguien critica a su hijo.
	Se articula con la Residencia en Medicina de Familia y Comunidad y los profesores del grado en Enfermería y Medicina.	Registro en el historial médico: Formulario de referencia rellenado y enviado al médico de referencia.

*EPA= Enfermería de Práctica Avanzada; ^†^CCU= Ciencia del Cuidado Unitario; ^‡^CIAP-2= Clasificación Internacional de Atención Primaria; ^§^PTS= Proyecto Terapéutico Singular; ^||^ TEA= Trastorno del Espectro Autista; ^¶^RAS= Red de Atención de Salud

**Figura 3:** Dibujo elaborado por M45 tras una intervención creada por la enfermera y estimulada por la frase: “Dibuja algo que represente a la mujer ideal”

## Discusión

Los resultados revelados en este estudio amplían el alcance de competencias que pueden desempeñar los enfermeros en la APS^14^ y ofrecen evidencia científica que respalda las prácticas de cuidado de enfermería orientadas al bienestar de la madre, con repercusiones positivas para los niños con TEA y otros miembros de la familia. En este escenario, los gestores e implementadores de políticas públicas pueden optar por la cualificación de enfermeros de la APS para el desarrollo e implementación de líneas de atención con el fin de aumentar las tasas de accesibilidad de los sistemas sanitarios universales, la capacidad de resolución de problemas de la APS, la reducción de desigualdades, la mejora de la calidad de vida y la reducción de costes^15^.

Muchos países carecen de programas de atención estructurados capaces de integrar un diagnóstico precoz, intervenciones multidisciplinares continuas y apoyo longitudinal para familias y personas con TEA^16^. Este escenario refuerza la necesidad de programas específicos coordinados por enfermeros, considerando que la mayoría de los modelos existentes se centran únicamente en la fase diagnóstica, sin garantizar un seguimiento adecuado o apoyo a las demandas emocionales y socioeducativas del núcleo familiar^17^.

En el momento de escribir este artículo, no existían programas ni líneas de atención a madres de niños con TEA, ni tampoco PAE basados en teorías de enfermería^18^, como el CCU. Por lo tanto, la gestión del cuidado en el CACTO la realiza la enfermera coordinadora del programa, quien fomenta el protagonismo de las madres, considerando el enfoque del cuidado, la evaluación, el diagnóstico y la prestación de la atención^9^.

El primer momento de desarrollo del CACTO se denomina “comprensión”^5^; se trata de comprender las necesidades de salud de las madres, por lo que es importante que las enfermeras accedan a una amplia variedad de literatura científica con altos niveles de evidencia y grado de recomendación, además de recopilar información esencial mediante visitas domiciliarias. Por lo tanto, antes de la implementación de la CACTO, considerando el subdominio centrado en la atención, se sabía que las madres necesitaban acceso a derechos de protección social, bienes y servicios como vivienda y saneamiento básico, seguridad alimentaria, trabajo y educación; servicios cualificados y atención multiprofesional empática; acogedor, fortalecimiento de lazos y aceptación; responsabilidad del padre y compartir las responsabilidades diarias y el autocuidado^5^.

El enfoque del cuidado en el CACTO se basa en la CCU, buscando la restauración curativa del ser vivo en función del equilibrio entre las dimensiones cuerpo-mente-alma que ocurre durante y después del cuidado transpersonal^7^. El enfermero que trabajaba en CACTO desarrolló un periodo de intercambio durante su doctorado, con el objetivo de dominar la CCU, una experiencia que contribuyó a planificar la aplicabilidad de los atributos de la APS durante la implementación del CACTO, como la territorialización, el vínculo, la participación social, la responsabilidad en salud y el protagonismo de los usuarios^18^.

Durante la anamnesis se exploró un diálogo amplio y exhaustivo para comprender mejor los riesgos y problemas que determinan el proceso de enfermedad de las madres. La concepción de las personas como sujetos históricos, constituida por las experiencias vividas que moldean sus creencias y comportamientos^19^, justifica la necesidad de llevar a cabo una anamnesis investigativa en profundidad, con la recopilación de datos desde el nacimiento hasta el momento presente, identificando traumas, tristezas, placeres y alegrías experimentados. En este momento, es oportuno valorar el proceso de trabajo del Agente Comunitario de Salud de referencia de la madre y su contribución a la elaboración del PTS^20^ de los usuarios.

La evaluación y el diagnóstico en la PAE es un proceso continuo que implica la recopilación y análisis de datos objetivos y subjetivos. Esto permite revisar diagnósticos, listas de problemas, planes de cuidado, intervenciones y estrategias basadas en el protagonismo del paciente y su red de apoyo^18^. En el proceso diagnóstico, la EPA debería utilizar datos de evaluación clínica atribuidos a sistemas estandarizados de clasificación clínica^21^ como el CIAP-2 explorado en el CACTO.

La EPA interesada en aplicar la CCU^7^ debe ejercer habilidades únicas como la capacidad de abstracción, la comprensión de la totalidad constitutiva del Ser humano sagrado, el reconocimiento de lo que se dice y lo que no se dice, de las expresiones y el silencio, el control de impulsos profesionales y personales que recogen los pensamientos de las personas en acogida, la creatividad en la creación de intervenciones (Figura 1) y acuerdos, así como agilidad para implementarlas en los momentos oportunos.

Los dilemas éticos que enfrentan las enfermeras en CACTO reservan complejidad que requiere procesos de toma de decisiones delicados y prudentes basados en las necesidades y prioridades de salud de las madres. Estas son experiencias entre separarse de la pareja y poner fin a situaciones de violencia o permanecer en la relación matrimonial por dependencia económica; o incluso, retomar sus objetivos profesionales y sentirse culpable por externalizar el cuidado del niño o seguir cuidándolo completamente y vivir con dificultades económicas. Las decisiones éticas de las enfermeras se basan en la legislación profesional, los protocolos institucionales y la Declaración Universal de Bioética y Derechos Humanos, además del principio de sensibilidad moral, que permite reconocer la dimensión ética de los problemas e implicar al paciente en el proceso de toma de decisiones^22^.

La colaboración interprofesional fomenta una cultura de respeto mutuo, confianza y reconocimiento por las contribuciones de cada miembro del equipo, creando un entorno propicio para la innovación, la creatividad y la excelencia en el servicio. Así, mejora el intercambio de conocimientos, mejora la atención y la toma de decisiones, aumentando la seguridad de la persona cuidada y optimizando los recursos^23^. El cuidado de las madres requiere el cumplimiento de las funciones de la APS como coordinadora de cuidados y organizadora de la RAS para optimizar el tiempo de respuesta, acortar los itinerarios terapéuticos para ellas y para los niños, y aumentar la capacidad de resolución de problemas basándose en las posibilidades de apoyo matricial con el equipo multidisciplinar en la APS (eMulti) y los terapeutas infantiles que conforman la RAS. El principio de intersectorialidad también debe ser coordinado por el equipo de la APS para atender las necesidades de salud de las madres con el Centro de Referencia de Asistencia Social (CRAS) y las escuelas/guarderías a las que asisten sus hijos.

Las necesidades de las madres se manifiestan como problemas emocionales, conductuales, psicosociales y clínico-biológicos que pueden entenderse como dominios del cuerpo, la mente y el alma^17^. La evaluación y el diagnóstico indican que la enfermera que trabaja en el CACTO debe explorar habilidades en la gestión de la atención de enfermedades crónicas; Por lo tanto, las acciones para prevenir riesgos y lesiones y promover la salud son prioritarias. La evidencia empírica relacionada con el cumplimiento de los acuerdos acordados en el CACTO apunta a una alta satisfacción de las madres, así como otros estudios incluidos en una revisión sistemática que indican que el desempeño de la EPA tiene un impacto positivo en la calidad de vida de los pacientes^24^.

El manejo de las enfermedades crónicas está guiado por la autovaloración, el reconocimiento de potencialidades y límites, la conciencia del propio proceso de salud-enfermedad, la alineación de las expectativas de curación, el grado de autodeterminación, el afrontamiento y la motivación^25^. Los atributos y funciones de la ESF, como la accesibilidad, el vínculo, la acogida, la humanización, la territorialización, la responsabilidad en salud, la coordinación y la longitudinalidad de la atención^26^ fueron elementos aplicados por la enfermera del CACTO y contribuyeron a revelar demandas ocultas de las madres, aumentar la tasa de asistencia a las consultas, promover la adhesión a los acuerdos implementados y favorecer la valorización social del papel materno y la autoestima.

La variedad de diagnósticos realizados y la característica única y personalizada del CACTO requirieron la implementación de un amplio ámbito de atención, así como la creación de nuevas curas basadas en la evidencia. La EPA debe desarrollar habilidades de capacidad investigadora para encontrar la mejor evidencia científica y esclarecer las preguntas que surjan en el campo de la práctica. Las competencias del ámbito de la investigación contribuyen a la innovación basada en el desarrollo de nuevas investigaciones, protocolos de promoción de la salud y la implementación de nuevas tecnologías de atención en la APS^9^.

También vale la pena destacar, basándose en esta experiencia, en la condición fundamental de protagonismo de los usuarios para el desarrollo de tecnologías culturalmente apropiadas. La diversidad y el amplio alcance de las competencias de los enfermeros del CACTO respaldan la relevancia de los cursos de nivel stricto sensu para mejorar los productos técnicos, fomentando el desarrollo de tecnologías sociales, la profundización epistemológica y la aplicabilidad de las teorías de enfermería, así como la gestión de programas de atención, la enfermería como ciencia y la implementación de la PAE en la APS.

Las enfermeras que trabajan en la APS deben presentar características como compromiso con la profesión, análisis crítico y reflexivo, capacidad de abstracción, habilidades educativas y clínicas consolidadas mediante formación cualificada en docencia e investigación. Estos aspectos les permitieron actuar clínicamente ante la variedad de diagnósticos, ofrecer diversidad de atención, coordinar planes de atención, promover la longitudinalidad de la atención, además de expresar autonomía y autoridad, cualidades esperadas por la enfermera practicante^14^.

Los resultados indican el fortalecimiento de la identidad profesional de la enfermera del CACTO, al mejorar su razonamiento clínico y su papel de liderazgo en el equipo interprofesional^27^, al proporcionar soluciones a cuestiones críticas aún no resueltas mediante la implementación de programas, políticas o prácticas destinadas a generar impactos en el acceso y las desigualdades para las personas en situaciones vulnerables. La evidencia de este estudio valora la calidad de la educación en enfermería, señala la relevancia de la APS y la necesidad de formación adicional en PAE en estudios de posgrado, con reservas respecto a las especificidades regionales de cada país.

Los resultados de un estudio de revisión de alcance revelaron que el desempeño de la EPA en la APS se asocia con menos visitas de pacientes a urgencias, menor incidencia de hospitalizaciones o readmisiones hospitalarias, menos consultas en la APS, mayor adhesión a los planes de atención y menor coste de los gastos relacionados con la salud^28^. La creación de la EPA a través de un curso doctoral en práctica de enfermería con énfasis en la APS es una iniciativa que se está expandiendo en todo el mundo^29^.

Una etnografía realizada con enfermeros de Suiza identificó las funciones de la EPA en la APS, a saber: definición de las funciones y funcionamiento del equipo de salud, resolución de conflictos interprofesionales, atención centrada en la persona, liderazgo colaborativo y comunicación interprofesional^30^. En el contexto internacional, la EPA ha contribuido a ampliar el acceso a la APS, ofreciendo atención favorable al envejecimiento activo y gestionando la atención de personas con enfermedades crónicas^31^.

En el Reino Unido, las EPA y las enfermeras de familia que trabajan en la comunidad desarrollan competencias similares, con mayor implicación de la EPA en la dimensión de investigación, mientras que los enfermeros familiares trabajan frecuentemente en los ámbitos de la educación y el liderazgo^32^. Sin embargo, varios estudios convergen en la comprensión de que las particularidades legales y las necesidades territoriales de cada país deben definir el ámbito de acción de la EPA^28^ ^-^ ^30^.

Este estudio contribuye al avance del conocimiento científico presentando iniciativas innovadoras para el cuidado de un público en amplio crecimiento y con necesidades sanitarias poco exploradas en los servicios de salud, así como madres de niños con TEA. Se entiende que las competencias adquiridas pueden apoyar a las enfermeras en una atención alineada con las necesidades de las madres. Los resultados nos permiten debatir las competencias desempeñadas por las enfermeras del CACTO y las aplicadas por las enfermeras de la APS en todo el mundo; por lo tanto, futuros estudios podrán examinar y evaluar la calidad de la atención para madres de niños con TEA y el proceso de formación de enfermeras de la APS, teniendo en cuenta las necesidades de salud de las madres.

Una limitación del estudio son las especificidades del instituto TEAbraco SAJ basado en los principios de asociativismo, voluntarismo y solidaridad, que pueden haber influido en los aspectos motivacionales, los procesos de toma de decisiones y las relaciones intersubjetivas entre enfermeras y madres. Por ello, la evidencia encontrada debe aplicarse con reservas en otros espacios de cuidado dentro de la RAS.

## Conclusión

Las competencias de la enfermera que trabaja en el CACTO se organizan en siete dimensiones: gestión del cuidado, ética, colaboración interprofesional, promoción y prevención, enfermería basada en la evidencia, investigación y liderazgo. Entre las competencias, destaca la creación e invención de nuevas formas de cuidado, la autonomía en el cuidado de las madres, la prudencia en dilemas éticos, la capacidad investigadora y de investigación, así como el liderazgo y el protagonismo para la coordinación del cuidado y organización de la RAS, con el fin de ampliar el ámbito de práctica en la APS y aumentar la resolución de problemas.

La enfermera que trabaja en el CACTO realizó competencias que pueden aplicar en la APS basándose en estrategias para ampliar el alcance de las prácticas de enfermería, como promover el autocuidado de las madres, contribuir al desarrollo psicosocial y cognitivo de los niños, responsabilizar a los padres y actuar políticamente a favor de la inclusión social de las familias de niños con TEA.

## Data Availability

Todos los datos generados o analizados durante este estudio están incluidos en este artículo publicado.
